# IL-1β, IL-23, and TGF-β drive plasticity of human ILC2s towards IL-17-producing ILCs in nasal inflammation

**DOI:** 10.1038/s41467-019-09883-7

**Published:** 2019-05-14

**Authors:** Korneliusz Golebski, Xavier R. Ros, Maho Nagasawa, Sophie van Tol, Balthasar A. Heesters, Hajar Aglmous, Chantal M. A. Kradolfer, Medya M. Shikhagaie, Sven Seys, P. W. Hellings, Cornelis M. van Drunen, Wytske J. Fokkens, Hergen Spits, Suzanne M. Bal

**Affiliations:** 1 Department of Experimental Immunology, Amsterdam UMC, University of Amsterdam, Location AMC, Meibergdreef 9, Amsterdam, 1105 AZ The Netherlands; 2 Department of Immunology and Microbiology, Lab of Clinical Immunology, KU Leuven, Belgium Herestraat 49-box 1030, BE-3000 Leuven, Belgium; 3 Department of Otorhinolaryngology, Amsterdam UMC, University of Amsterdam, Location AMC, Meibergdreef 9, Amsterdam, 1105 AZ The Netherlands

**Keywords:** Inflammation, Innate lymphoid cells, Mucosal immunology

## Abstract

Innate lymphoid cells (ILCs) are crucial for the immune surveillance at mucosal sites. ILCs coordinate early eradication of pathogens and contribute to tissue healing and remodeling, features that are dysfunctional in patients with cystic fibrosis (CF). The mechanisms by which ILCs contribute to CF-immunopathology are ill-defined. Here, we show that group 2 ILCs (ILC2s) transdifferentiated into IL-17-secreting cells in the presence of the epithelial-derived cytokines IL-1β, IL-23 and TGF-β. This conversion is abrogated by IL-4 or vitamin D3. IL-17 producing ILC2s induce IL-8 secretion by epithelial cells and their presence in nasal polyps of CF patients is associated with neutrophilia. Our data suggest that ILC2s undergo transdifferentiation in CF nasal polyps in response to local cytokines, which are induced by infectious agents.

## Abstract (web summary)

Innate lymphoid cells (ILCs) play critical immunological roles including immune surveillance at mucosal sites. Here the authors show that during nasal inflammation pathogen-induced cytokine production guides the differentiation of ILCs.

## Introduction

Cystic fibrosis (CF) is a life-limiting multisystem genetic disorder caused by mutations in the CF transmembrane conductance regulator (*CFTR*) gene^1^, a cAMP-regulated epithelial chloride channel^2^. The major consequences of CFTR mutations are abnormally viscous secretions in the upper and lower airways, impaired mucociliary clearance, persistent infection, and chronic inflammation^3^. Consequently, the respiratory tract of most patients with CF becomes infected with opportunistic bacteria, most importantly *Staphylococcus aureus* (SA) and *Pseudomonas aeruginosa* (PA)^4^. This eventually results in lung damage and loss of pulmonary function, the major cause of morbidity and mortality in CF disease^5^. Notably, chronic rhinosinusitis with nasal polyps (CRSwNP) is frequent in CF. Up to 40% of CF patients will manifest NP in their lifetime^6^, whereas CRSwNP prevalence in the general population is estimated at 2–5%^7^.

Despite massive neutrophil infiltration and excessive production of pro-inflammatory cytokines, CF patients display persistent and difficult to treat bacterial infections of the upper and lower airways^8^. Recent reports provide evidence that targeting specific inflammatory pathways reduce the exuberant inflammatory response that compromises the host’s ability to control infection^9^. IL-1β, which is involved in lung inflammation, promotes PA-induced early neutrophil recruitment in the lung via a synergistic interaction with IL-23^10^. Thus, IL-1β, which is present in sputum of CF patients in high amounts, could be a potent target to ameliorate the pathogenic consequences of the microbial colonization in CF^11^. Furthermore, IL-1β triggers the activation of pathogenic IL-17A-secreting T cells, highly enriched in CF patients^12,13^. Anti-IL-17 therapy significantly suppresses neutrophil recruitment and inflammation in CF mouse models after PA infection^14^. Besides Th17 cells, the type 3 subset of innate lymphoid cells (ILC3) is a major producer of IL-17^15^. This raises the question whether these strategically located cells contribute to the cytokine milieu in CF.

ILCs are involved in early stages of the immune host defense mounted against pathogens and mediate the generation of lymphoid organs and tissue remodeling^16^. ILCs subsets have been identified based on their cytokine profiles and by the expression of key transcription factors that regulate their development and function^17^. ILC1s produce IFN-γ and need the Tbox transcription factor family member T-bet; ILC2s require the GATA-3 transcription factor and produce IL-4, IL-5, and IL-13, and ILC3s produce IL-17, IL-22, GM-SCF, and TNF-α and express RORγt. Strategically located at the body’s interface with the environment, ILCs promptly respond to pathogenic threats by secreting effector cytokines and igniting adaptive immune responses in synchrony with myeloid cells^18^. ILCs are activated by cytokines and chemokines secreted by triggered epithelial cells or other cells of the immune system^19^. Several studies in humans and mice have now established that ILCs are highly plastic, adapting their cytokine secretion profiles in response to changes in the microenvironment. Thus, ILC3s and ILC2s can transdifferentiate into IFN-γ-producing ILC1s in the presence of IL-12 and ILC1s can shift into ILC3s in response to IL-23, IL-1β, and retinoic acid^20–22^. These transdifferentiation processes result in increased IFN-γ producing ILC1-like cells as observed in inflamed tissue of Crohn’s patients and in lung tissue from individuals with severe COPD^22,23^.

Previously we reported that ILC2s accumulate in NP of patients suffering from CRS^24,25^. Here we observed that IL-17-producing ILCs predominate in NP from CF patients. Unexpectedly, we found that majority of these IL-17-producing ILCs are derived from ILC2s which transdifferentiated to IL-17-producing cells by the epithelium-derived cytokines IL-1β, IL-23, and TGF-β. We present evidence that these ILC contribute to the increase of neutrophils which mediate the inflammation in NP of CF patients.

## Results

### ILC3s accumulate in nasal polyps from CF patients

**Fig. 1:**
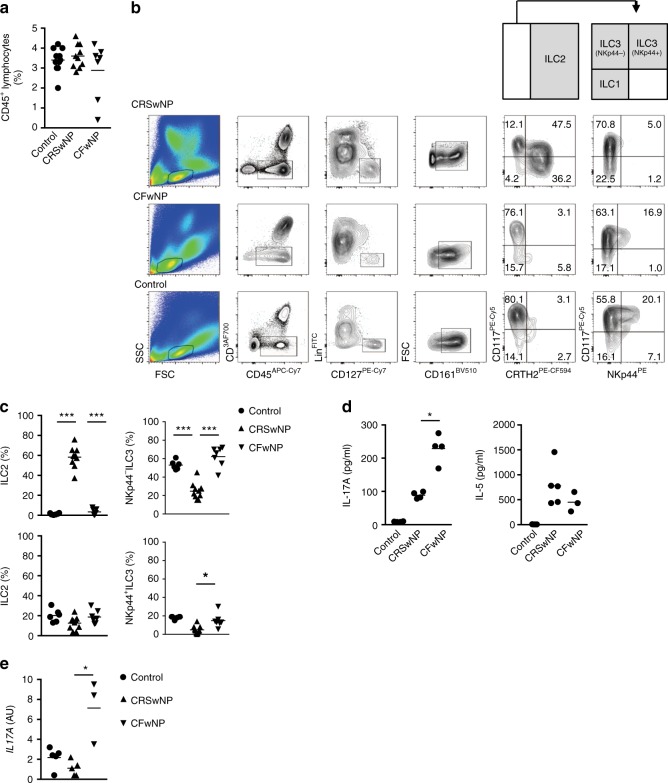
ILC3s accumulate in nasal polyps from CF patients. **a** Frequency of total ILCs in nasal tissues of healthy subjects (*n* = 10), CRSwNP (*n* = 11), and CFwNP subjects (*n* = 7). **b** Gating strategy for flow cytometric analysis of human ILCs in nasal tissues, with ILCs defined as: Lin^−^ (CD1a, CD3, CD14, CD16, CD19, CD34, CD94, CD123, BDCA2, FcεRI, TCRαβ, TCRγδ), CD45^+^ CD127^+^CD161^+^ cells. From this population CRTH2^+^ cells define ILC2s; CRTH2^−^CD117^−^NKp44^−^ cells define ILC1s, and CD117^+^ cells define ILC3s. NKp44^+^ further subdivides two subpopulations of ILC3s. **c** ILC subtypes distribution in healthy nasal tissue inferior turbinate (*n* = 6), CRSwNP (*n* = 10), and CFwNP (*n* = 7). Each symbol represents an individual donor and the horizontal lines represent the mean. **d** IL-17A and IL-5 levels in homogenized nasal tissues of healthy (*n* = 4–5), CRSwNP (*n* = 4–5), and CFwNP (*n* = 3–4). Data are normalized to the total protein concentration in each sample. **e** *IL17A* gene expression in CD117^+^NKp44^−^ ILC3s isolated from nasal tissues of healthy (*n* = 5), CRSwNP (*n* = 5), and CFwNP subjects (*n* = 3). Data are normalized to the expression of GAPDH. Data are presented as individual values (**a**, **c**–**e**) of two to four independent experiments, *p < 0.05, **p < 0.01, ***p < 0.001 as determined by one way ANOVA. Source data

To evaluate the role of ILCs in the sinonasal inflammation in CF patients, we examined the composition of ILC subsets in NP of these patients as compared with those from CRSwNP patients and healthy nasal tissue (inferior turbinates) (patient characteristics in Supplementary Fig. 1a). Although the total frequency of ILCs in these three tissues were similar (Fig. 1a), the composition differed. A significant increase in the proportion of NKp44^−^ ILC3s was found in CFwNP as compared with CRSwNP. (Fig. 1b, c). In contrast, ILC2s predominate in CRSwNP patients (Fig. 1b, c) and were almost undetectable in NP from CF patients and inferior turbinates. The CD117^+^NKp44^−^CRTH2^−^ ILCs from CFwNP did not express KLRG1, CD25 and had low ICOS expression (Supplementary Fig. 1b). We did observe significant GATA-3 expression in CD117^+^NKp44^−^CRTH2^−^ ILCs from CFwNP, but not in CD117+NKp44^−^CRTH2^−^ ILCs from CRSwNP patients (Supplementary Fig. 1b). Even though the ILC composition in NP from CF patients and healthy turbinate was similar, only in the inflamed tissue inflammatory cytokines could be detected (Fig. 1d). NP from CF patients displayed a significant increase in IL-17A when compared with NP from CRS patients whereas IL-5 levels were similar. Moreover, ILC3s from CFwNP patients produced IL-17A, whereas ILC3s and ILC2s from healthy turbinate tissue or from NP of CRS patients did not (Fig. 1e). Although we cannot exclude that Th17 cells and other cells can also be a source of IL-17 in these patients, this indicates that NKp44^−^ ILC3s are at least partly responsible for the IL-17 production in NP of CF patients.

### Nasal epithelialium derived cytokines drive ILC2 plasticity

**Fig. 2:**
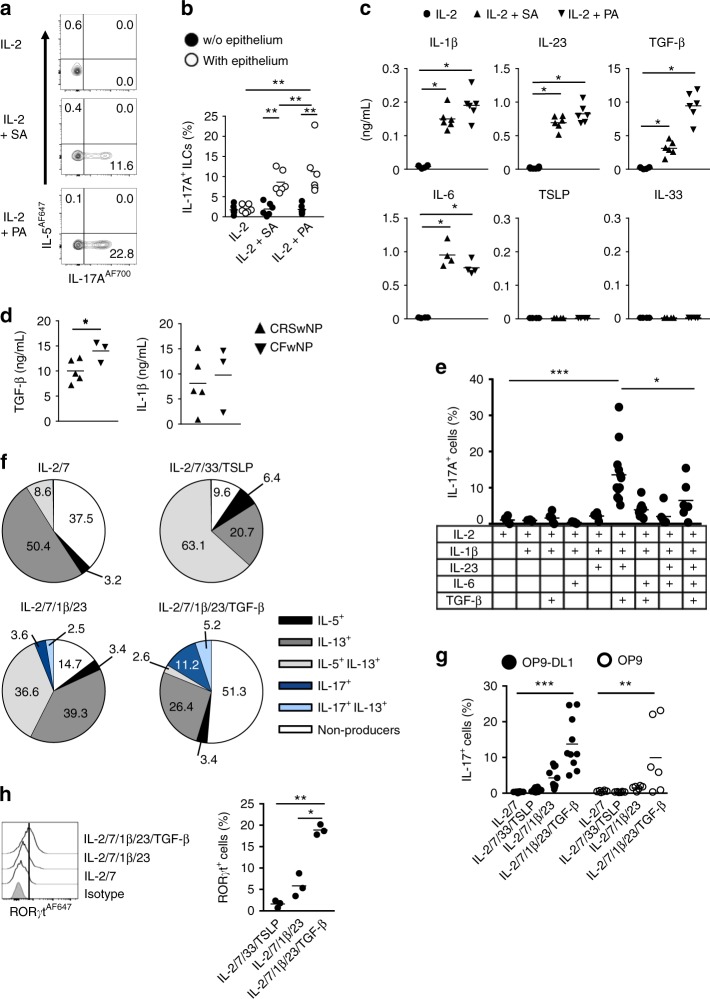
Nasal epithelial derived cytokines drive ILC2 plasticity. **a** Representative flow cytometric analysis of intracellular IL-5 and IL-17A expression after PMA/ionomycin stimulation of blood-derived ILC2s cultured with NP epithelium exposed to SA or PA for 7 days. **b** Quantification of IL-17A-producing ILC2s after stimulation as in **a** (*n* = 6). **c** Quantification of IL-1β, IL-23, TGF-β (*n* = 6), and IL-6, TSLP and IL-33 (*n* = 4) in supernatants of NP epithelium exposed for 24 h to IL-2, IL-2 + PA, or IL-2 + SA. **d** TGF-β and IL-1β in homogenized NP tissues of CRS patients with (*n* = 3) or without CF (*n* = 5). Each symbol represents an individual donor and the horizontal lines represent the mean. Data are normalized to the total protein concentration in each sample. **e** Quantification of intracellular IL-17A-producing ILC2s upon 5–7 days exposure to IL-2 plus IL-1β and additionally IL-23, TGF-β, or IL-6 or a combination of these cytokines (*n* = 6). **f** Quantification of intracellular cytokine-producing ILC2s exposed to IL-2 (20 U/mL) and IL-7 (and all other cytokines 20 ng/mL); IL-2, IL-7, IL-33, and TSLP; IL-2, IL-7, IL-1β, and IL-23; IL-2, IL-7, IL-1β, IL-23, and TGF-β for 8 days on the OP9-DL1 cell line after PMA/ionomycin restimulation. **g** Quantification of IL-17A-producing blood-derived ILC2s upon culture as in **f** on OP9 or OP9-DL1 cells. **h** Representative flow cytometric analysis (left panel) and quantification (right panel) of RORγt expression by blood ILC2s cultured for 8 days with IL-2 and IL-7; IL-2, IL-7, IL-1β, and IL-23; IL-2, IL-7, IL-1β, IL-23, and TGF-β on the OP9-DL1 cell line (*n* = 3). Data are presented as individual values (**b**–**e**,** g**, **h**) or as mean (**f**) of two to five independent experiments; *p < 0.05, **p < 0.01, ***p < 0.001 as determined by one way ANOVA or Student’s *t* test. Source data

Our findings raised the question of why NP from CF patients do not contain ILC2s whereas in morphologically similar NP from CRS patients ILC2s predominate. One possibility is that the cytokine microenvironment in these polyps is different, resulting in altered ILC compositions. If correct, we would predict that the microenvironment in CF is disadvantageous and that of CRS advantageous for ILC2s. Indeed, we published previously that NP from CRS patients contain IL-4 and TSLP that strongly stimulate ILC2s^22^. Given that CF patients suffer from infections with SA or PA, we hypothesized that these pathogens might be responsible for a reduction of ILC2s observed in NP from these patients. Therefore, we cultured peripheral blood ILC2s directly with these pathogens or with NP epithelial cells (from CRS patients) that were exposed to SA or PA. We did not observe a reduction of ILC2s due to cell death, but surprisingly, coculture of SA or PA-exposed epithelial cells with ILC2s resulted in an increase of IL-17A, without production of IL-5 by these cells (Fig. 2a, b), suggesting that these bacteria induced transdifferentiation of ILC2s to IL-17A-producing ILCs. ILC2s did not respond to the bacteria in the absence of epithelial cells. Culturing of blood-derived ILC1s or CD117^+^CRTH2^−^ ILCs with SA or PA exposed epithelial cells did not result in IL-17A production (Supplementary Fig. 2a). We reasoned that cytokines derived from epithelial cells drive IL-17A production by ILC2s. Infection with SA-induced or PA-induced high production of IL-1β, IL-23, and TGF-β in nasal epithelial cells, whereas no IL-33 or TSLP could be detected (Fig. 2c). IL-1β is a general activator of ILC2s and important for allowing ILC2 to ILC1 transdifferentiation^22,26^. Both in NP from CRS and CF patients, high levels of IL-1β were detected. We could not detect IL-23, but TGF-β was significantly increased in NP from CF as compared with those from CRS patients (Fig. 2d). These cytokines are implicated in Th17 polarization^15,27^ and indeed, the combination of these three cytokines induced potent IL-17A production by proliferating ILC2s (Fig. 2e, Supplementary Fig. 2b, c). Additionally, exposure of nasal epithelium to SA or PA induced high production of IL-6 (Fig. 2c), a cytokine which has also been implicated in Th17 polarization in vitro^28^. However, IL-6 did not contribute to the transdifferentiation of ILC2s into IL-17-producing ILCs, but instead reduced this process (Fig. 2e, Supplementary Fig. 2f).

### TGF-β is involved in transdifferentiation of ILC2

It was previously reported that CD117^+^CRTH2^−^ ILCs from blood cultured on the stromal cell line OP9 in the presence of IL-2, IL-7, IL-1β, and IL-23 were able to differentiate into multiple ILCs linages, including IL-17-producing ILCs^23^. We analyzed the progeny of bulk ILC2s cultured in similar culture settings to exclude that the observed IL-17 production was due to the differentiation of precursor cells contaminating the ILC2 population. ILC2s that were cultured on the stromal cells with IL-2 and IL-7 produced IL-13 and the addition of either IL-33 and TSLP or IL-1β and IL-23 potently induced IL-5 and further promoted IL-13 production (Fig. 2f, Supplementary Fig. 2d, e). Whereas culture with IL-1β and IL-23 resulted in little IL-17A expression, supplementation of TGF-β drastically increased IL-17A production and inhibited IL-5 production by ILC2s (Fig. 2f, g, Supplementary Fig. 2c, d, e). Accordingly, culture of ILC2s with IL-2, IL-7, IL-1β, IL-23, and TGF-β resulted in a significant induction of RORγt (Fig. 2h). Notch signaling was shown to play a role in promoting the plasticity of ILC2s^29^. We observed that the frequency of IL-17A-producing cells was similar in the absence or presence of DL1 (Fig. 2g) indicating that Notch-1 signaling is not involved in transdifferentiation of ILC2s to IL-17-producing cells.

### ILC2s give rise to IL-17 producing cells at a clonal level

**Fig. 3:**
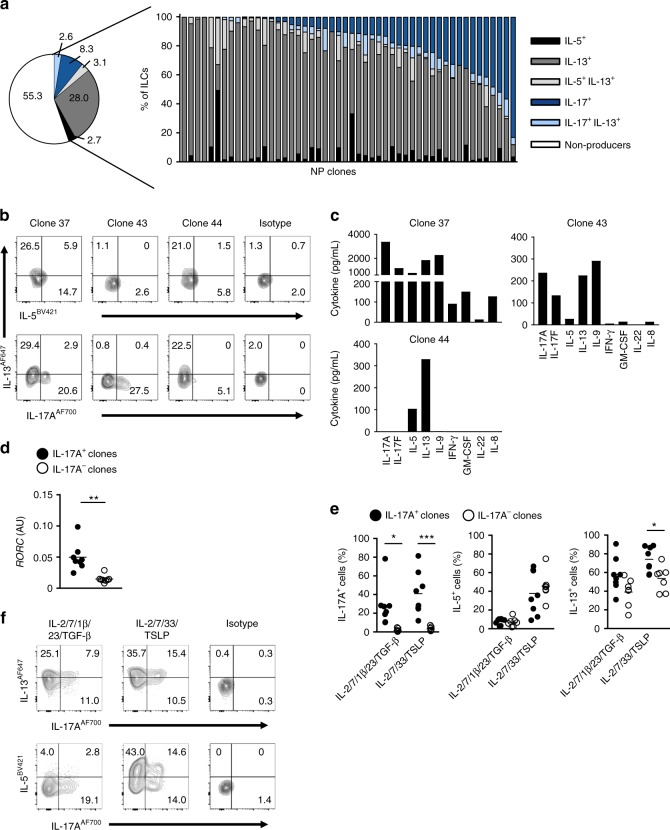
ILC2s give rise to IL-17 producing cells at a clonal level. **a**–**d** Single ILC2s from CRS NP were sorted by FACS into 96-well round bottom plates pre-seeded with OP9-DL1 and stimulated with IL-2 (20 U/mL), IL-7, IL-1β, IL-23, and TGF-β (20 ng/mL each). **a** After 14-21 days, cultures were analyzed for intracellular cytokine production (IL-5, IL-13, and IL-17A) after PMA/ionomycin restimulation. **b** Representative flow cytometric analysis of intracellular cytokine production of three clones. **c** Quantification of concentration of cytokines of three clones. **d** *RORC* expression in IL-17A^+^ and IL-17A^−^ clones (*n* = 8 per group). Each symbol represents an individual clone and the horizontal lines represent the mean. **e**, **f** Clonal cultures were resorted and cultured for 5 days on OP9-DL1 with IL-2, IL-7, IL-1β, IL-23, and TGF-β; or IL-2, IL-7, IL-33, and TSLP. **e** Quantification of intracellular IL-5, IL-13, and IL-17A production after PMA/ionomycin stimulation (*n* = 7–8 clones per group). Each symbol represents an individual donor and the horizontal lines represent the mean. **f** Representative flow cytometric analysis of cytokine production of an IL-17^+^ clone. Data are presented as individual values (**c**–**e**) of three independent cloning experiments with one donor each. *p < 0.05, **p < 0.01, ***p < 0.001 as determined by one way ANOVA or Student’s *t* test. Source data

Next, we analyzed the progeny of single ILC2s derived from NP from CRS patients or blood in the presence of IL-2, IL-7, IL-1β, IL-23, and TGF-β (Fig. 3a, Supplementary Fig. 3a). With a cloning efficiency of 5–22%, we were able to grow clones within 14 days of culture on OP9 stromal cells. Almost all clones gave rise to IL-13-producing progeny, while a substantial part of each clone was producing IL-17A, similar to the inflammatory ILC2s (iILC2s) that have been described in mice^30^ (Fig. 3a, Supplementary Fig. 3a). A minor part still produced IL-5. There was a lot of variation in the fraction of IL-17A-producing cells that was obtained from each clone, with some clones exclusively producing IL-17A, whereas other clones gave rise to IL-5, IL-13, and IL-17A producing cells. **(**Fig. 3b, c), suggesting that within the progeny of one clone cells with different cytokine secretion profiles developed. PB ILC2 clones were more potent in generating IL-17-producing cells as compared with NP ILC2s. Comparison of IL-17A-producing with non-IL-17A-producing clones revealed that the former ones had a higher *RORC* expression (Fig. 3d). When the progeny of IL-17A-producing clones were cultured in the presence of IL-33 and TSLP, these cells regained the ability to produce IL-5 to a similar extent as the progeny from non-IL-17A-producing clones (Fig. 3e, f, Supplementary Fig. 3b). At the same time, these cells retained their IL-17A production following stimulation with TSLP and IL-33. In summary, these data indicate that ILC2s are a heterogeneous population and that a fraction of ILC2s can transdifferentiate into-IL-17A-secreting cells, whereas part of these cells remains resilient to conversion. The capacity of ILC2s to produce IL-17A is, at least in some cases, a stable feature.

### The tissue cytokine balance modulates ILC2 plasticity

**Fig. 4:**
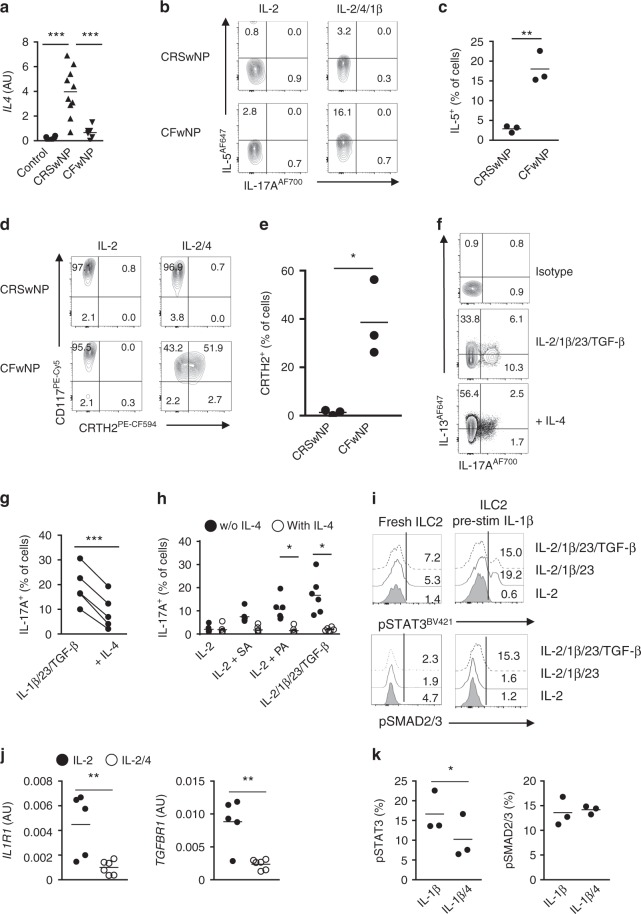
The tissue cytokine balance modulates ILC2 plasticity. **a** Expression of *IL4* transcripts in nasal tissues of inferior turbinates (*n* = 6), NP from patients with CRS (*n* = 10), and CF (*n* = 6). **b** Representative flow cytometric analysis of IL-5 and IL-17A production by CD117^+^CRTH2^−^NKp44^−^ ILCs from CFwNP and CRSwNP after culture for 5–7 days with IL-2 or IL-2, IL-4, and IL-1β. **c** Quantification of IL-5-producing cells (*n* = 3 per group). **d** Representative flow cytometric analysis of CRTH2 expression on CD117^+^CRTH2^−^NKp44^−^ ILCs from CFwNP and CRSwNP after culture for 5–7 days with IL-2 or IL-2 plus IL-4. **e** Quantification of CRTH2-expressing cells (*n* = 3 per group). **f** Representative flow cytometric analysis of intracellular IL-5 and IL-17A production by blood ILC2s upon culture for 5–7 days with IL-2, IL-7, IL-1β, IL-23, and TGF-β in the absence or presence of IL-4. **g** Quantification of IL-17A-producing cells (*n* = 5). **h** Quantification of IL-17A-producing ILCs after coculture with NP epithelial cells upon 5-7 days stimulation with IL-2; IL-2, PA; IL-2, SA; IL-2, IL-1β, IL-23, and TGF-β with and without IL-4. ILC2s were pre-exposed for 4 days to IL-4 or not (*n* = 6). **i** Representative flow cytometric analysis of phosphorylation of STAT3 and SMAD2/3 upon activation with IL-2; IL-2, IL-1β, and IL-23; or IL-2, IL-1β, IL-23, and TGF-β in freshly isolated blood ILC2s or after priming for 7 days with IL-2 and IL-1β. **j** Expression of *IL1RL1* and *TGFBR1* transcripts in blood ILC2s upon culture for 4 days with IL-2 or IL-2 and IL-4 (*n* = 5–6). **k** Quantification of phosphorylation of STAT3 and SMAD2/3 upon activation with IL-2, IL-1β, IL-23, and TGF-β in blood ILC2s that were primed for 7 days with IL-2, IL-1β; or IL-2, IL-1β, and IL-4 (*n* = 3). Data are presented as individual values with mean (**a**, **c**, **e**, **g**, **h**, **j**, **k**) from 3 to 4 independent experiments. *p < 0.05, **p < 0.01, ***p < 0.001 as determined by one way ANOVA or Student’s *t* test. Source data

The observation that the ILC composition in NP of CF or CRS patients differed, prompted us to evaluate the presence of distinct inflammatory cytokines in the local nasal tissues that can modulate the process of plasticity. Whereas TGF-β protein was increased in CF as compared with CRS (Fig. 2d), IL-4 gene was only found in CRSwNP tissue (Fig. 4a). Since we have previously shown that IL-4 is able to revert the IL-12-mediated transdifferentiation of ILC2s into ILC1s^22^, we investigated whether the absence of IL-4 alters the ILC composition in NP of CF patients. We isolated CD117^+^CRTH2^−^NKp44^−^ ILCs from CFwNP patients and activated these cells in the presence of IL-4 and IL-1β. Indeed, under these conditions, CD117^+^CRTH2^−^NKp44^−^ ILCs from CF patients acquired the ability to secrete IL-5 (Fig. 4b, c). Furthermore, IL-4 alone upregulated CRTH2 in part of the CD117^+^CRTH2^−^NKp44^−^ ILCs (Fig. 4d, e). In contrast, CD117^+^CRTH2^−^NKp44^−^ ILCs isolated from CRSwNP patients did not become ILC2s in the presence of IL-4. We assessed whether IL-4 also affected the ILC2 into ILC3 conversion induced by IL-1β, IL-23, and TGF-β. Culturing of ILC2s with IL-1β, IL-23, and TGF-β in the presence of IL-4 inhibited ILC2 transdifferentiation into IL-17A-producing cells (Fig. 4f, g). Transdifferentiation of ILC2s was prevented as well when IL-4 pre-stimulated ILC2s were cocultured with SA or PA infected epithelial cells (Fig. 4h).

We next investigated the mechanism underlying the plasticity process. IL-1β is known to induce activation and cytokine responsiveness of ILC2s^31^ and indeed pre-stimulation with IL-1β made the cells responsive to IL-23 by STAT3 phosphorylation (Fig. 4i). Similar STAT3 phosphorylation was observed in the presence or absence of TGF-β, but TGF-β addition induced additional phosphorylation of SMAD2/3, indicating that additional activation of this pathway is probably necessary to induce optimal expression of RORγt and IL-17^32^. IL-4 inhibition of the transdifferentiation is accompanied by downregulating the *TGFBR1* and *IL1R1* gene expression (Fig. 4j) and inhibition of STAT3 phosphorylation (Fig. 4k) indicating that IL-4 makes ILC2s unresponsive to IL-1β and TGF-β.

### Transcriptome analysis of ILC2 plasticity

**Fig. 5:**
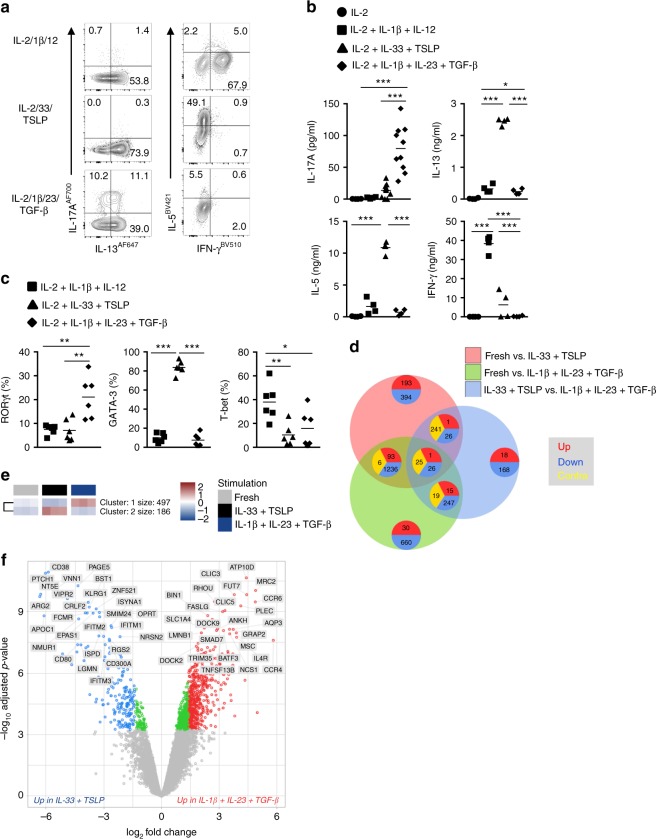
Transcriptome analysis of ILC2 plasticity. **a** Flow cytometry of intracellular IL-5, IL-13, IL-17A, and IFN-γ production after restimulation with PMA/ionomycin of blood ILC2s exposed to IL-2; IL-2, IL-1β, and IL-12; IL-2, IL-33, and TSLP; IL-2, IL-1β, IL-23, and TGF-β for 5-7 days. **b** Quantification of the concentration of IL-5, IL-13, IL-17A, and IFN-γ produced by ILC2s cultured as in **a** (*n* = 4–10). Each symbol represents an individual donor and the horizontal lines represent the mean. **c** Quantification of RORγt-expressing, GATA-3-expressing, and T-bet-expressing blood ILC2s upon exposure to IL-2, IL-1β, and IL-12; IL-2, IL-33, and TSLP; IL-2, IL-1β, IL-23, and TGF-β for 5-7 days by intracellular staining. Each symbol represents an individual donor and the horizontal lines represent the mean (*n* = 6). **d** Venn diagram showing the overlap of the number of genes that are significantly up-regulated (red), down-regulated (blue), or contra-regulated (yellow). (red circle) freshly isolated ILC2s versus ILC2s cultured with IL-2, IL-33, and TSLP. (blue circle) freshly isolated ILC2s versus ILC2s cultured with IL-2, IL-1β, IL-23, and TGF-β. (green circle) ILC2s cultured with IL-2, IL-33, and TSLP versus ILC2s cultured with IL-2, IL-1β, IL-23, and TGF-β. **e** Summary of unbiased clustering. Clusters of the number of genes with significant different expression between blood ILC2s stimulated as in **d**. **f** Vulcano plot comparing ILC2s cultured with IL-2, IL-33, and TSLP versus ILC2s cultured with IL-2, IL-1β, IL-23, and TGF-β. The 25 most significantly up-regulated or down-regulated genes are labeled. Data are presented as individual values with mean of two independent experiments (**b**). *p < 0.05, **p < 0.01, ***p < 0.001 as determined by one way ANOVA. Source data

We demonstrated previously that ILC2s can transdifferentiate to IFN-γ-producing ILC1s^22^. Here, we investigated the cytokine potential of differentiating ILC2s in more detail. Blood-derived ILC2s were cultured in the presence of IL-2, IL-1β, and IL-12; IL-2, IL-33, and TSLP; or IL-2, IL-1β, IL-23, and TGF-β as compared with unstimulated ILC2s (cultured with low dose IL-2 only). As reported before, ILC2s that upon stimulation with IL-2, IL-33, and TSLP produce IL-4, IL-5, IL-9, and IL-13, respond to IL-2, IL-1β, and IL-12 by secreting IFN-γ, whereas production of the type 2 cytokines is inhibited (Fig. 5a, b, Supplementary Fig. 4a). Activation with IL-2, IL-1β, IL-23, and TGF-β selectively increased IL-17A, IL-17F, and IL-9 (Fig. 5a, b, Supplementary Fig. 4a). A subset of ILC2s co-expressed IL-13 and IL-17 upon stimulation with IL-2, IL-1β, IL-23, and TGF-β. (Supplementary Fig. 4b). Irrespective of the type of stimulation, ILC2s potently produced IL-8 (Supplementary Fig. 4a). Consistent with the cytokine profile, RORγt, GATA-3, and T-bet were selectively increased by stimulation with IL-2, IL-1β, IL-23, and TGF-β; IL-2, IL-33, and TSLP; or IL-2, IL-1β, and IL-12, respectively, indicating that ILC2 conversion was accompanied by changes in expression of transcription factors (Fig. 5c, Supplementary Fig. 4c).

To further study the functional and phenotypic consequences of ILC2 plasticity, we compared the transcriptome of ILC2s in response to cytokine stimulations that result in differentiation into IL-5 or IL-17-producing cells. Both stimulations resulted in many genes being differentially expressed as compared with freshly isolated ILC2s (Fig. 5d, Supplementary Dataset 1) and all three groups formed distinct clusters (Supplementary Fig. 4d). Further analysis was done to identify specific expression patterns for IL-5 and IL-17-producing ILC2s. We identified two distinct clusters of genes that were specifically expressed upon stimulation with either IL-33 plus TSLP, or IL-1β plus IL-23 and TGF-β (Fig. 5e, Supplementary Fig 5). Cluster 1, consisting of 497 genes (*p* values < 0.01; a 2log change in expression of over 1.4 fold) that are enriched upon stimulation with IL-1β, IL-23, and TGF-β, among which ILC3 and Th17 related genes such as *RORC* and *CCR6* (Supplementary Fig. 5). *CCR6* was also among the top 25 of most significantly expressed genes as compared with cells stimulated with IL-33 and TSLP (Fig. 5f). Cluster 2 contained 186 genes that are enriched upon stimulation with IL-33 and TSLP, including ILC2 and Th2 related genes such as *IL1RL1*, *IL5*, or *IL13* (Supplementary Fig. 5). The most significantly differentially expressed genes included known ILC2 receptors such as *KLRG1* and *CRLF2* (which encodes the TSLP receptor) (Fig. 5f, Supplementary Fig. 4f).

**Fig. 6:**
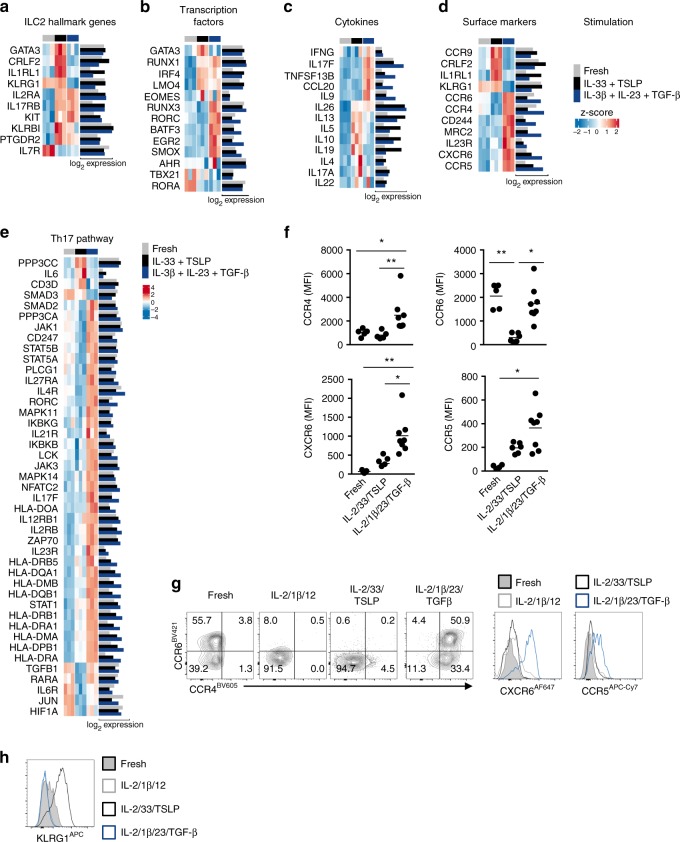
ILC2 stimulations result in unique expression patterns. **a** Heatmap of genes related to ILC2 signature. **b** Heatmap of genes related to ILC-related transcription factors. **c** Heatmap of genes related to ILC-related cytokines. **d** Heatmap of genes encoding significantly differentially expressed surface markers **d**. Both z-scores (row-normalized) and absolute expression values are shown. **e** Heatmap of genes significantly different expressed in ILC2s upon stimulation with IL-2, IL-1β, IL-23, and TGF-β as compared with ILC2s cultured with IL-2, IL-33, and TSLP that are related to the Th17 KEGG pathway. **f** Quantification of expression of CCR4, CCR6, CCR5 and CXCR6 on freshly isolated blood ILC2s, and upon culture for 5 days with IL-2, IL-33 and TSLP or IL-2, IL-1β, IL-23, and TGF-β (*n* = 5-8). **g** Representative flow cytometric analysis of CCR4, CCR6, CCR5, and CXCR6 upon culture as in **f**. **h** Representative flow cytometric analysis of KLRG1 expression upon culture as in **f**. Data are presented as individual values with mean of two to four independent experiments (**f**). *p < 0.05, **p < 0.01 as determined by one way ANOVA. Source data

Many genes encoding markers that have been described to be specific for ILC2s are affected by stimulation (Fig. 6a). Blood ILC2s are naïve cells as the expression of most ILC2 markers, such as *KLRB1, IL1RL1, CRLF2, PTGDR2, IL17RB*, and *GATA3* was increased upon stimulation with IL-33 and TSLP. Only elevated expression of *IL1RL1* and *CRLF2* was specific for IL-5/IL-13-producing cells, whereas expression of the other markers was also elevated upon induction of IL-17 production, suggesting that, similar to mouse iILC2s, human ILC2s that are induced by IL-1β, IL-6, IL-23, or TGF-β are still responsive to IL-25^30^. Expression of *RORC* was in concordance to what we observed after intranuclear staining with a RORγt-specific antibody (Figs. 5c, 6b), whereas *AHR* was highly expressed in freshly isolated ILC2 and remained high upon stimulation. IL-17-producing ILCs also displayed higher expression of the ILC3 related transcription factors *RUNX3, EGR2, BATF3* and *SMOX*^33,34^ (Fig. 6b). Neither type of stimulation induced *TBX21* nor *EOMES*.

As expected, *IL5* and to a lesser extend *IL13* were specifically induced by IL-33 and TSLP, whereas stimulation with IL-1β, IL-23, and TGF-β increased expression of *IL17F*, but also of *IL9*, *CCL20*, and *TNFSF13B* (encoding BAFF) (Fig. 6c). Some IL-9 production was induced by IL-33 and TSLP, however IL-1β, IL-23, and TGF-β induced significantly higher IL-9 production (Supplementary Fig. 4a). Stimulation with IL-33 and TSLP resulted in expression of *IL10* and *IL19*, whereas both type 2 and type 3 stimulations induced *IL26* expression (Fig. 6c). In contrast to *IL17F*, we did not observe an increase of *IL17A* transcripts in ILC2s stimulated for 6 days with IL-1β plus IL-23, and TGF-β compared with unstimulated ILC2 (Fig. 6c), even though transcripts of *RORC*, that controls both IL-17A and IL-17F expression, were increased and secretion of IL-17A protein was specifically induced (Fig. 5a, b). This may be due to mRNA instability or to the kinetics of induction of IL-17A transcripts. KEGG pathway analysis revealed that many differentially expressed genes that were selectively upregulated upon stimulation with IL-1β plus IL-23, and TGF-β belong to the Th17 pathway (Fig. 6e).

A number of surface markers were selectively upregulated upon stimulation with either IL-1β, IL-23, and TGF-β; or IL-33 and TSLP (Fig. 6d). IL-17-producing ILC2s expressed chemokine receptors that were reported to be expressed on Th17 cells such as *CCR6*, *CCR4*, and *CXCR6*^35,36^ suggesting that IL-17-producing ILC2s are migratory cells as also described for mouse iILC2^37^. We observed increased expression of the co-inhibitory SLAM family member *CD244*, which is also expressed on NK and CD8^+^ T cells and the mannose receptor *MRC2*, which can be induced on hepatocytes by TGF-β^38^. Upregulation of *CCR6, CCR4, CXCR6*, and *CCR5* gene expression upon stimulation with IL-1β, IL-23, and TGF-β also led to increased protein expression as was confirmed by staining with specific antibodies (Fig. 6f, g). We observed that whereas a subset of blood ILC2s expressed CCR6, this expression was retained on IL-17-producing ILC2s, but lost upon stimulation with IL-33 and TSLP or IL-1β and IL-12 (Fig. 6g). CD117^+^CRTH2^−^NKp44^−^ ILCs from blood, which include ILC precursors, did contain CCR6^+^ cells, but these cells did not co-express CCR4 nor CXCR6 (Supplementary Fig. 6a). The CCR6^+^ IL-17-producing ILCs resemble the MHCII expressing ILC3 subpopulation found in human tonsils (Supplementary Fig. 6b)^39^. In mice, these CCR6^+^ MHCII^+^ ILC3s regulate CD4^+^ T cells in the intestine^40^. Stimulation with IL-1β, IL-23, and TGF-β also resulted in decreased expression of a cluster of 15 genes, including *PTGER2* (encoding the prostaglandin E2 receptor), which ligand was shown to be a suppressor of ILC2 activation (Supplementary Fig. 6c)^41^. KLRG1, which is expressed on blood ILC2s and maintained by IL-33 and TSLP, was also downregulated by IL-1β, IL-23, and TGF-β (Fig. 6h).

### Transdifferentiated ILC2s enhance neutrophil recruitment

**Fig. 7:**
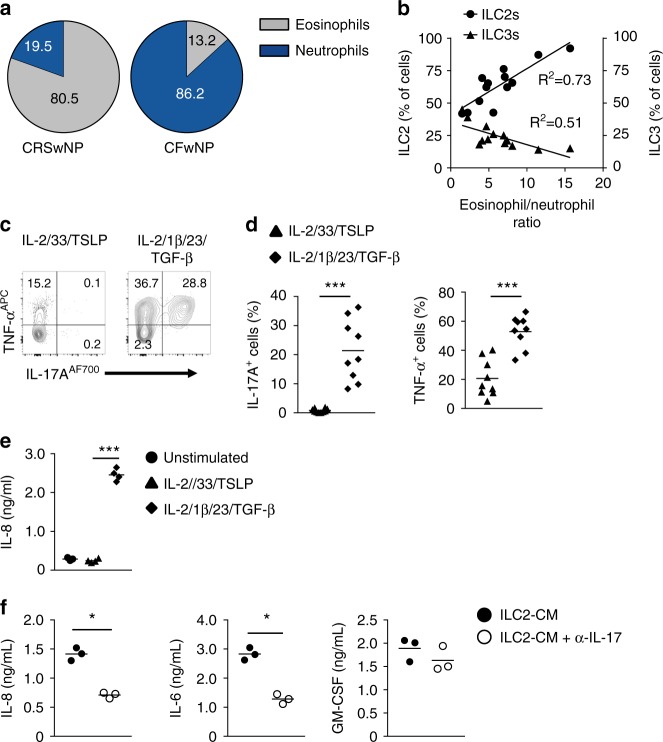
Transdifferentiated ILC2s enhance neutrophil recruitment. **a** Quantification of ractions of eosinophils and neutrophils in NP tissues of subjects with (*n* = 5) or without (*n* = 6) CF as determined by flow cytometry. **b** Correlation of ILC2 and NKp44^-^ILC3 frequencies to eosinophil/neutrophil ratio in CRSwNP (*n* = 13). **c**, **d** Representative flow cytometry analysis (**c**) and quantification (**d**) of intracellular expression of IL-17A and TNF-α in blood ILC2s exposed to IL-2, IL-33, and TSLP or IL-2, IL-1β, IL-23, and TGF-β for 5-7 days after PMA/ionomycin (*n* = 9–10). **e** Quantification of IL-8 production by NCI-H292 epithelial cells after 48 hr exposure to conditioned medium of ILC2s that were stimulated with IL-2, IL-33, and TSLP; or IL-2, IL-1β, IL-23, and TGF-β for 5–7 days (*n* = 3–4). **f** Quantification of IL-8, IL-6, and GM-CSF production by NCI-H292 epithelial cells in the presence or absence of IL-17-blocking antibody upon culture as described in **e** (*n* = 3). Data are presented as individual values with mean of two to four independent experiments (**d**–**f**). *p < 0.05, **p < 0.01, ***p < 0.001 as determined by one way ANOVA or Student’s *t* test

Whereas NP of CRS patients are characterized by an eosinophilic inflammation, the granulocyte population within CFwNP patients consist mainly of neutrophils (Fig. 7a)^42^. Analysis of ILCs and granulocytes in NP from CRS and CF patients revealed a positive correlation between ILC2s and the eosinophil to neutrophil ratio in NP, whereas the proportions of CD117^+^NKp44^−^ ILC3s negatively correlated with this ratio (Fig. 7b) suggesting that ILCs are involved in the recruitment of eosinophils and neutrophils. Consistent with this notion is our previous observation that ILC2s in CRSwNP activate eosinophils^22^. Here we found that IL-1β, IL-23, and TGF-β induced secretion of IL-17A and TNF-α (Fig. 7c, d), which are potent activators of epithelial cells^43^. We observed that stimulation of epithelial cells with supernatant from ILC2s activated with IL-1β, IL-23, and TGF-β induced strong IL-8 production by epithelial cells (Fig. 7e). This was not due to the remaining IL-2, IL-1β, IL-23, and TGF-β in these supernatants as these cytokines did not directly activate epithelial cells (Supplementary Fig. 7a). The IL-8 production by these epithelial cells could be partially blocked by adding anti-IL-17A antibody to the conditioned medium (Fig. 7f), indicating that IL-17A secreted by ILC2s is partly responsible for IL-8 secretion by epithelial cells. IL-1β, IL-23 and TGF-β also induced production of IL-8 and in addition GM-CSF which can contribute to neutrophil recruitment (Supplementary Fig. 7b). Since stimulation with IL-1β, IL-23 and TGF-β inhibits IL-5 production by ILC2s it is likely that transdifferentiated ILC2s are responsible for the low eosinophil to neutrophil ratio in CFwNP.

### Vitamin D3 prevents ILC2 conversion

**Fig. 8:**
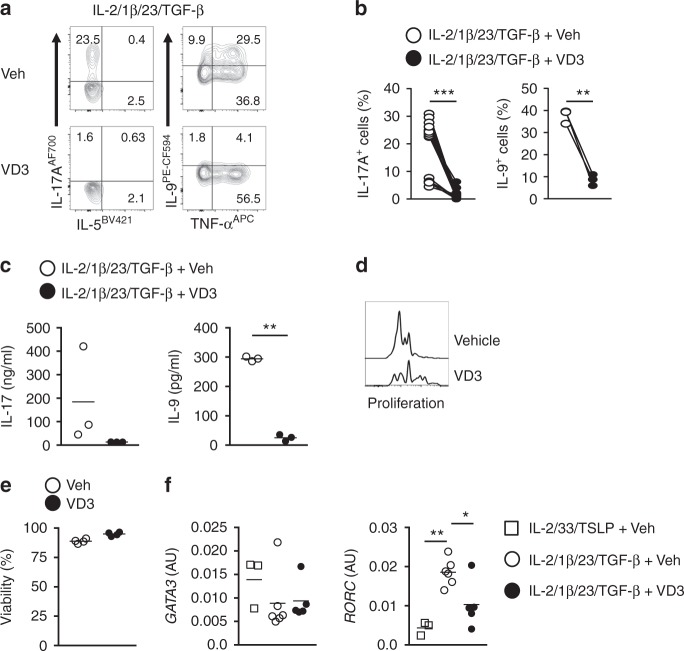
Vitamin D3 prevents ILC2 conversion by dampening IL-23 signaling pathway. **a** Representative flow cytometric analysis of intracellular IL-5, IL-17A, TNF-α and IL-9 production by blood ILC2s after culture for 7 days with IL-2, IL-1β, IL-23, and TGF-β in the presence of VD3 or vehicle control. **b** Quantification of IL-17A and IL-9-producing cells after culture as in **a** (*n* = 3–13). **c** Quantification of concentration of IL-17A and IL-9 after culture as in **a** (*n* = 3). **d** Representative flow cytometric analysis of ILC2 proliferation after culture as in **a**. **e** Quantification of viability of ILC2s after culture as in **a**. **f** Expression of *GATA3* and *RORC* transcripts in blood ILC2s upon culture for 7 days with IL-2, IL-33 and TSLP in the presence of vehicle; or IL-2, IL-1β, IL-23 in the presence of VD3 or vehicle control (*n* = 3–6). Data are presented as individual values with mean of two to four independent experiments (**b**, **c**, **e**, **f**). *p < 0.05, **p < 0.01, ***p < 0.001 as determined by one way ANOVA or Student’s *t* test. Source data

Vitamin D3 (VD3) insufficiency is common in CF patients despite VD3 supplementation^44^. Low serum VD3 levels are associated with poor lung function, more frequent exacerbations, and neutrophilia in asthma patients^45^. Moreover, VD3 deficiencies have been associated with high IL-17A and TNF-α production^46^. Given that ILC2-derived IL-17A and TNF-α may contribute to neutrophilia in CF, we investigated whether VD3 might block ILC2 plasticity towards IL-17A-secreting cells. VD3 strikingly reduced the production of IL-17A and IL-9 by IL-1β, IL-23, and TGF-β, whereas TNF-α production was not affected (Fig. 8a, b, c). VD3 partly inhibited cell proliferation (Fig. 8d) without affecting cell viability (Fig. 8e). IL-5 production upon stimulation with IL-5-promoting cytokines was not affected (Supplementary Fig. 8a,b). VD3 inhibits upregulation of RORγt induced by IL-1β, IL-23, and TGF-β (Fig. 8f) consistent with the demonstration that VD3 downregulates the IL23R expression on ILC3s^47^. Altogether, these results indicate that VD3 prevents the conversion of ILC2s into IL-17-producing ILCs.

## Discussion

CRSwNP is a disease of the upper airways characterized by tissue eosinophilia, high IL-5 and IgE, Th2-polarized responses, and an enrichment of ILC2s. We and others have previously shown that ILC2s are enriched in NP from CRS patients^24,22^ and that their frequencies correlate with the allergic state^48^, and eosinophilia^49^. In the present study, we observed that the ILC composition in NP of CF patients is very different, despite morphological and clinical similarities with NP of CRS subjects. In NP of CF patients, the frequency of ILC2s was diminished, whereas that of NKp44^−^ ILC3s was increased, as compared with NP of CRS patients. These ILC3s produce IL-17 and together with Th17 cells, which are also enriched in NP of CF patients, may be responsible for the increased levels of IL-17 these inflamed nasal tissues^42^. Whether the same holds true in the lungs of CF patients remains to be confirmed. ILC3s can be divided into different subsets based on the expression of NCRs (NKp44 and NKp46). Earlier findings indicate that in a number of tissues NKp44^−^ ILC3s produce IL-17A whereas NKp44^+^ ILC3s produce IL-22^20,50,51^. Also in CF NP, NKp44^−^ ILC3s were found to produce IL-17A, but our data strongly suggest that these cells are derived from ILC2s that do not produce IL-22 or IFN-γ. Next to transdifferentiated ILC2s, ILC precursors that specifically develop into IL-17-producing ILC3s can also contribute to the observed IL-17 production.

ILC plasticity is governed by cytokines and mediators present within the local tissue environment and ILC transdifferentiation has been reported in the context of chronic inflammatory diseases. We and others previously reported that IL-1β and IL-12 drive plasticity of ILC2s towards IFN-γ-producing ILC1-like cells which was associated with COPD^21,22^. Here, we demonstrate that stimulation of ILC2s with a combination of IL-1β, IL-23, and TGF-β, produced in high quantities by epithelium infected with SA or PA, resulted in an upregulation of RORγt, the signature transcription factor of ILC3s, downregulation of GATA-3 and production of IL-17. Whereas IL-1β by itself is a potent activator of ILC2s and induces the production of IL-5 and IL-13^22,26^, it acts synergistically with IL-23 and TGF-β to induce production of IL-17 by ILC2. These cells also produce GM-CSF and IL-8 which, together with epithelial cell-produced IL-8 induced by IL-17, add to the neutrophil promoting environment in NP of CF patients. IL-1β levels are elevated in sputum of CF patients^52^, which is consistent with the notion that IL-1β, by potentiating ILC2 activation and facilitating plasticity^31^, is a major factor in inflammation in CF polyps. Further, a recent study demonstrated the presence of IL-17-producing ILC2s within the skin lesions of psoriasis patients (Bernink et al., submitted to Nat. Immunology) supporting the idea that ILC2-ILC3 plasticity may play a pathogenic role in chronic diseases associated with IL-17.

The finding that IL-4 completely inhibits the conversion of ILC2s to IL-17-producing ILCs provides an explanation for the striking difference in ILC composition in NP in CRS as compared with CF. IL-4, as well as another ILC2 stimulator TSLP, are highly expressed in NP of CRS patients and consequently inhibit transdifferentiation of ILC2s into IL-17-producing cells. In the present study, we find that NP of CF patients do not contain IL-4. The absence of IL-4 may create an environment that promotes plasticity of residing ILC2s. In CRS, there is an accumulation of eosinophils and ILC2s supported by an amplifying loop of IL-4-producing eosinophils and IL-5-producing ILC2s^22^, whereas in CF, the ILC2s stimulate recruitment of neutrophils via IL-17 and IL-8.

VD3 deficiency is correlated with more severe inflammation of the nasal mucosa in CRSwNP patients^53^ and associated with the presence of NP in CF^54^. Recently, it was shown that VD receptor-deficient mice accumulate IL-22-producing ILCs in their intestines and that these mice are more susceptible to colitis^55^. In the present study, we show that VD3 selectively blocks IL-17A, IL-9 production, and RORC expression by ILC2s exposed to IL-1β, IL-23, and TGF-β. VD3 has been shown to regulate Th17 responses as VD receptor knockout mice have elevated Th17 cells and IL-17 production^56^. Our findings are consistent with observations that downregulation of the IL-23 receptor upon exposure to VD3 plays a critical role in modifying the IL-17 pathway in mucosal ILC3s^47^. Further identification of modulators of ILC function may be of interest for the design of therapeutic interventions, especially those targeting the downstream or upstream pathways that regulate ILC activation and recruitment^57^. Current treatment options for CF aim to improve the airway function and prevent microbial infection by physical removal of mucus combined with anti-microbial and anti-inflammatory agents, however new therapeutic strategies include CFTR correctors that are designed to improve defective protein processing, trafficking and cell surface expression and potentiators that increase the activity of mutant CFTR at the cell surface^58^. Specific targeting of IL-17 combined with anti-microbial treatment might result in a reduced neutrophilia and severity of inflammation in the lower and upper airways. The increase in airway IL-17 in CF is most likely due to a dysregulated inflammatory response to infection which subsequently results in a massive influx of neutrophils. In a *cftr*^−/−^ mouse model the importance of IL-17 in the pathology of PA-induced airways inflammation was demonstrated^14^. The neutralization of IL-17 prior to infection significantly improved the outcomes in the CF mice, suggesting IL-17 as a therapeutic target. This is supported by the reduced airway tissue damage and burden of infection in an *Il17a*^−/−^ model of PA infection^59^. Notably, in the *ctfr*^−/−^, *il1r1*^−/−^ d/d mouse model of PA infection, neutralization of IL-1β attenuated the infection and improved bacterial clearance^10^. This suggests that IL-1β could be another interesting target for controlling the CF pathology.

ILC2 transdifferentiation into IL-17A-producing ILC2s required TGF-β to block IL-5 production and induce RORγt and IL-17 production in a SMAD2 dependent manner. Upon stimulation with IL-1β, IL-23, and TGF-β we generated cells that co-produce IL-13 and IL-17, similar to the iILC2s that have been described in mice^30,37^. iILC2s are recruited from the intestines in response to IL-25 or to an infection with *N. brasiliensis*. In mice, iILC2s can be identified by the expression of KLRG1, CCR9, and IL-25R, while lacking the IL-33R. Here we show that in humans, both IL-5-producing ILC2s and IL-17-producing iILC2 express the IL-25R, with the latter lacking the IL-33R. In contrast to mouse iILC2s, human IL-17-producing ILC2s lack KLRG1 and CCR9. In mice, iILC2s are derived from an intestinal precursor and are able to differentiate into natural IL-5 and IL-13-producing ILC2s in vivo^60^. The nature of the intestinal ILC2 precursor in mice is unknown. Our findings suggest that IL-17-producing iILC2s can be derived from mature nILC2s, although we cannot exclude the existence of a common ILC2 precursor for IL-17-producing ILC2s and nILC2s in humans as well. The IL-17-producing cells we describe here are very different from the recently described murine ILC2s that produce IL-5, IL-13, and IL-17 in an AHR-dependent, but RORyt-independent manner in response to IL-33^61^. These cells retain their ILC2 characteristics and are also very different from the iILC2s as they express similar levels of ST2 as nILC2s and do not depend on IL-25. An interesting fundamental difference between iILC2s and nILC2s in mice is that iILC2s are migratory cells whereas nILC2s are tissue resident^37,62^. Whether or not the IL-17-producing human ILC2s are migratory cells is unknown, but it is noteworthy that they express chemokine receptors, including CCR4, that are not expressed on nILC2s.

In summary, our findings demonstrate that the functional activities of ILC2s are strongly influenced by the microenvironment. Plasticity permits tissue resident ILC2s to adapt to changing conditions and may also change their migratory properties. Consequently, ILC2 transdifferentiation will facilitate the eradication of invading pathogens that require different types of immune responses or pathogens that colonize multiples niches along their life cycle. Henceforward, upon disease remission, ILCs might undergo cell-reprograming to avoid excessive mucosal damage, restore the mucosal tissue, and re-establish a beneficial interplay with commensal flora.

## Methods

### Tissue collection

All tissues were collected after subjects provided informed consent in accordance with approved protocols by the Medical Ethical Committee of the Academic Medical Center in Amsterdam, the Netherlands. Uninflamed nasal inferior turbinates were obtained from patients who underwent corrective surgery for hypertrophy with or without septoplasty. Nasal polyps were obtained from CRSwNP patients with or without cystic fibrosis undergoing endoscopic sinus surgery. Buffy coats were provided by the blood bank at Sanquin, Amsterdam.

### Isolation of cells

Nasal tissues were manually cut into fine pieces and digested for 45 min at 37 °C with Liberase TM (125 µg/mL) and DNase I (50 U/mL). Cell suspensions were filtered through 70 µm nylon strainer and pelleted by centrifugation (5 min, 400 × *g*). Peripheral blood mononuclear cells (PBMCs) were isolated by Lymphoprep (Stemcell Technologies) density gradient centrifugation. Epithelial cells were obtained by incubating single cell suspension with anti-EpCAM MicroBeads (Miltenyi Biotec) and a positive selection on a magnetic column.

### Flow cytometry sorting and analysis

For sorting cells from PBMCs, cells were first enriched by labeling with PE-conjugated anti-CD161, followed by anti-PE microbeads (Miltenyi) according to the manufacturer’s instructions. Sorting from nasal tissues was performed without pre-enrichment. Cells were stained and sorted using the following monoclonal (all anti-human) antibodies (clone, catalog number) Lin^-^: (FITC-conjugated CD1a (HI149, 300104), CD3 (OKT3, 317306), CD14 (HCD14, 325604), CD16 (3G8, 555406), CD19 (HIB19, 302206), CD34 (581, 2317520), CD94 (DX22, 305504), CD123 (H6H, 306014), BDCA2 (201A, 354208), FcεRI (AER37, 2273040), TCRαβ (IP26, 306706), TCRγδ (B1, 331208)), AF700-conjugated CD45^+^ (HI30, 304024), PE-Cy7-conjugated CD127^+^ (R.3434, A64618), and PE-conjugated CD161^+^ (HP3G10, 339904). Cells were additionally stained with PE-Cy5-conjugated CD117 (104D2D1, B96754). PE-CF594-conjugated CRTH2 (BM16, 3450126) and PE-conjugated NKp44^+^ (P448, 325108) were used to sort CRTH2+ ILC2s and CD117^+^ CRTH2^−^ NKp44^+^ ILC3s and CD117^+^ CRTH2^−^ NKp44^−^ ILCs using a FACSAria (BD) to a purity >99% either in bulk or as single cells.

Granulocytes were identified as follows: neutrophils: SSC^high^ AF700-conjugated CD3^−^ (UCHT1, 300424), APC-conjugated HLA-DR^−^ (L243, 307609), FITC-conjugated CD123^−^ (H6H, 306014), APC-Cy7-conjugated CD16^+^ (3G8, 302017); eosinophils: SSC^high^CD3^−^HLA-DR^-^CD123^−^CD16^−^ and PE-Dazzle-conjugated Siglec-8^+^ (7C9, 304109).

For experiments involving intracellular cytokine staining, cells were stimulated with PMA (10 ng/mL; Sigma) plus Ionomycin (500 nM; Merck) in the presence of Golgi Plug (BD) for 3 h at 37 °C. Afterwards cells were fixed, permeabilized, and stained using the Foxp3/Transcription Factor Staining Buffer Kit (ThermoFisher Scientific). For detection of pSTAT3 and pSMAD2/3 samples were stimulated for 10 min. Cells were fixed (BD Cytofix fixation buffer, BD Biosciences) for 15 min at 4 °C and permeabilized (BD Phosflow Perm Buffer III) for 30 min on ice. Afterwards, cells were stained with antibodies for 30 min at room temperature. Samples were acquired on LSRFortessa or FACSCanto II (BD Biosciences) and analyzed with FlowJo software (TreeStar).The following antihuman antibodies were used: FITC-conjugated: IL-17F (Poly5166, 3183029); PE-conjugated: IL-9 (MH9A4, 507605), IL-5 (JES1-39D107, 500904), GATA3 (TWAJ, 12996642); AF700-conjugated: IL-17A (BL168, 512318); BV421-conjugated: IL-5 (JES1-39D107, 504311), STAT3-Phospho (13A31, 651009); BV510-conjugated: IFN-γ (4SB3, 502544); APC-conjugated: IL-13 (JES10-5A2, 501903), RORγT (Q21559, 563620), KLRG1 (13F12F2, 17948842), Smad2/Smad3 (072670, 562696); PE-Cy7-conjugated: Tbet (4B10, 1282582). All antibodies were used at 1:200 dilution and were purchased from Biolegend, eBioscience, or Beckman Dickinson.

### Experimental setup

Lin^-^CD127^+^CD161^+^ ILC populations were cultured for 5 to 7 days in Yssel’s medium (made ‘in house’, Academic Medical Center, Amsterdam), supplemented with 1% heat inactivated human AB serum (Merck), and 1% penicillin and streptomycin (Roche). 3000–5000 cells were stimulated for 5-7 days with IL-2 (10 U/mL) with combinations of IL-33, TSLP, IL-4, IL-1β, TGF-β, and IL-23 (all at a concentration of 50 ng/mL). In some experiments, VitD3 (50 nM, Merck) or ethanol vehicle were added.

For some bulk and all cloning experiments, cells were cultures on the murine stromal cell line OP9-DL1 (3000 cells were pre-seeded in 96-well round bottom plates one night before coculture). For bulk cultures (500–1000), ILC2s were stimulated for 8–10 days with IL-2 (20 U/mL), IL-7 (20 ng/mL), and various combinations of IL-33, TSLP, IL-1β, TGF-β, IL-23, and IL-4 (all at a concentration of 20 ng/mL). For bulk culture, fresh cytokines were added after 5 days. For cloning experiments, cytokines and medium were replenished once per week and cells were analyzed after 2–3 weeks.

Nasal epithelial cells were grown in 75 mL culture flasks in Bronchial Epithelial Cell Growth Medium (Lonza) until confluency or in PneumaCult-ALI medium (Stemcell Technologies) in the air-liquid interface (ALI) model. For ALI, epithelial cells were seeded onto permeable membrane of culture inserts. The basal epithelial cell surface was in contact with liquid culture medium, whereas the apical surface was exposed to air. Upon differentiation, cells were exposed to PA or SA (500,000 of bacteria cells/mL) for 5–7 days. At the same time, ILC2s (3000–5000) were added to the culture medium.

NCI-H292 cells were grown in RPMI 1640 medium (Gibco) supplemented with 10% FCS (ThermoFisher Scientific). For culturing with ILC2 conditioned medium, ILC2s were stimulated for 7 days in RPMI 1640 with IL-2 (10 U/mL) in combination with either IL-33 and TSLP, or IL-1β, IL-23, and TGF-β (all at a concentration of 50 ng/mL). NCI-H292 cells (3000 cells/well) were pre-seeded in 96-well flat bottom plates. In some experiments, anti-IL-17 (5 µg/mL, R&D Systems) or IgG2B isotype was added.

IL-5, IL-13, IL-17A, IL-8, IL-6, and GM-CSF were measured in supernatants by enzyme-linked immunosorbent assay (ThermoFisher Scientific). Multiple cytokines were detected in some experiments by U-plex technology (Meso Scale Diagnostics).

### Quantitative real-time PCR

Total RNA was extracted with a NucleoSpin RNA XS kit (Macherey-Nagel) according to the manufacturer’s instructions. cDNA was synthesized with a High-Capacity cDNA Reverse Transcription kit (ThermoFisher Scientific). PCRs were performed in BioRad iCycler (BioRad) with IQ SYBR Green Supermix (Bio-Rad,) using the primers described in Supplementary Table 1. Bio-Rad CFX Manager 3.1 software was used for quantification of expression. All samples were normalized to the expression of control genes encoding GAPDH and β-actin and results are presented in arbitrary units. The sequences of primers used in this study are collected in the Supplementary Table 1.

### Microarray analysis

To isolate total RNA, sorted cells were flash frozen in PBS immediately after sorting and stored at −80 °C prior to RNA extraction. QIAzol Lysis Reagent (Qiagen) was added to the cells, and RNA was isolated and purified using the RNeasy kit (Qiagen). The concentration was measured on a NanoDrop ND-2000 (Thermo Scientific) and RNA integrity was examined using the 2200 TapeStation System with Agilent RNA ScreenTapes (Agilent Technologies). Total RNA was amplified using the GeneChip WT Pico Kit (Thermo Fisher Scientific) generating biotinylated sense-strand DNA targets. The labeled samples were hybridized to human Clariom D arrays (Thermo Fisher Scientific). Washing and staining was performed by the GeneChip Fluidics Station 450 and the scanning was performed using the GeneChip Scanner 3000 (both Thermo Fisher Scientific). All cell populations were generated in triplicate. All data analysis was performed in RStudio. Raw data were normalized using the RMA algorithm implemented in the limma R-package. Adjusted *p*-values were calculated using the *Benjamini-Hochberg* method. Data were visualized using ggplot2 and pheatmap R-packages.

### Statistical analysis

Statistical significance was determined with ANOVA, Student’s *t*-test, or Mann–Whitney *U*-test. GraphPad Prism software was used.

### Reporting summary

Further information on research design is available in the Nature Research Reporting Summary linked to this article.

## Supplementary information

Supplementary Information

Description of Additional Supplementary Files

Supplementary Data 1

Reporting Summary

Source Data

## Data Availability

The datasets generated during and/or analyzed during the current study are available from the corresponding author on reasonable request. The source data underlying Figs. 1a, c–e, 2b–i, 3a, c–e, 4a, c, e, g, h, j, k, 5b, c, 6f, 7a, b, d–f, 8 b, c, e, f are available in the Source Data file. The microarray data reported in this paper has been uploaded to Gene Expression Omnibus (GEO) with the accession number GEO: GSE124926 and log2 expression values of all genes are available in Supplementary Dataset 1.
