# Supplementary material for: IL-1β, IL-23, and TGF-β drive plasticity of human ILC2s towards IL-17-producing ILCs in nasal inflammation
Source: Nat Commun. 2019 May 14;10:2162. doi: 10.1038/s41467-019-09883-7 (PMC6517442; doi:10.1038/s41467-019-09883-7)
Supplement: Supplementary file 1 — Supplementary Information [file 41467_2019_9883_MOESM1_ESM.pdf]

Supplementary information

Golebski et al.: IL-1beta, IL-23, and TGF-beta drive plasticity of human ILC2s towards IL-17-producing ILCs in nasal inflammation

Supplementary Figure 1. (related to Figure 1)

A

	Healthy control	CRSwNP	CFwNP
N	11	13	7
Age	41 (21-55)	49 (20-62)	20 (18-27)
M/F ratio	41/59	55/45	43/57
Allergy	2	3	2
Asthma	0	1	0

B

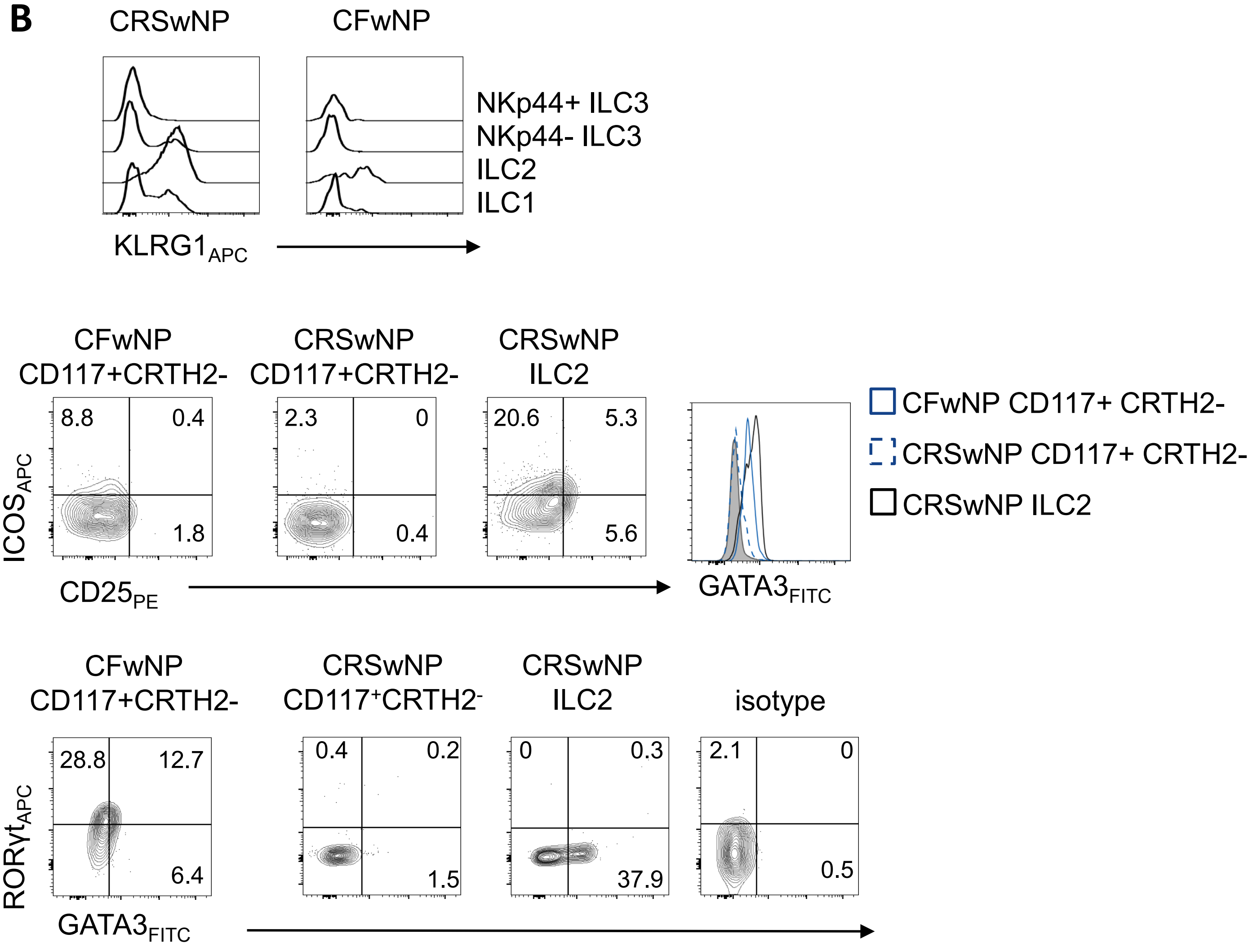

Supplementary Figure 1. ILC3s accumulate in nasal polyps from CF patients.
(A) Patients characteristics (age, sex, atopic status, asthma). (B) Representative flow cytometric analysis of KLRG1, ICOS, CD25, GATA-3, and RORγt expression on nasal polyp ILCs from CRS and CF patients (n = 3).

Supplementary Figure 2. (related to Figure 2)

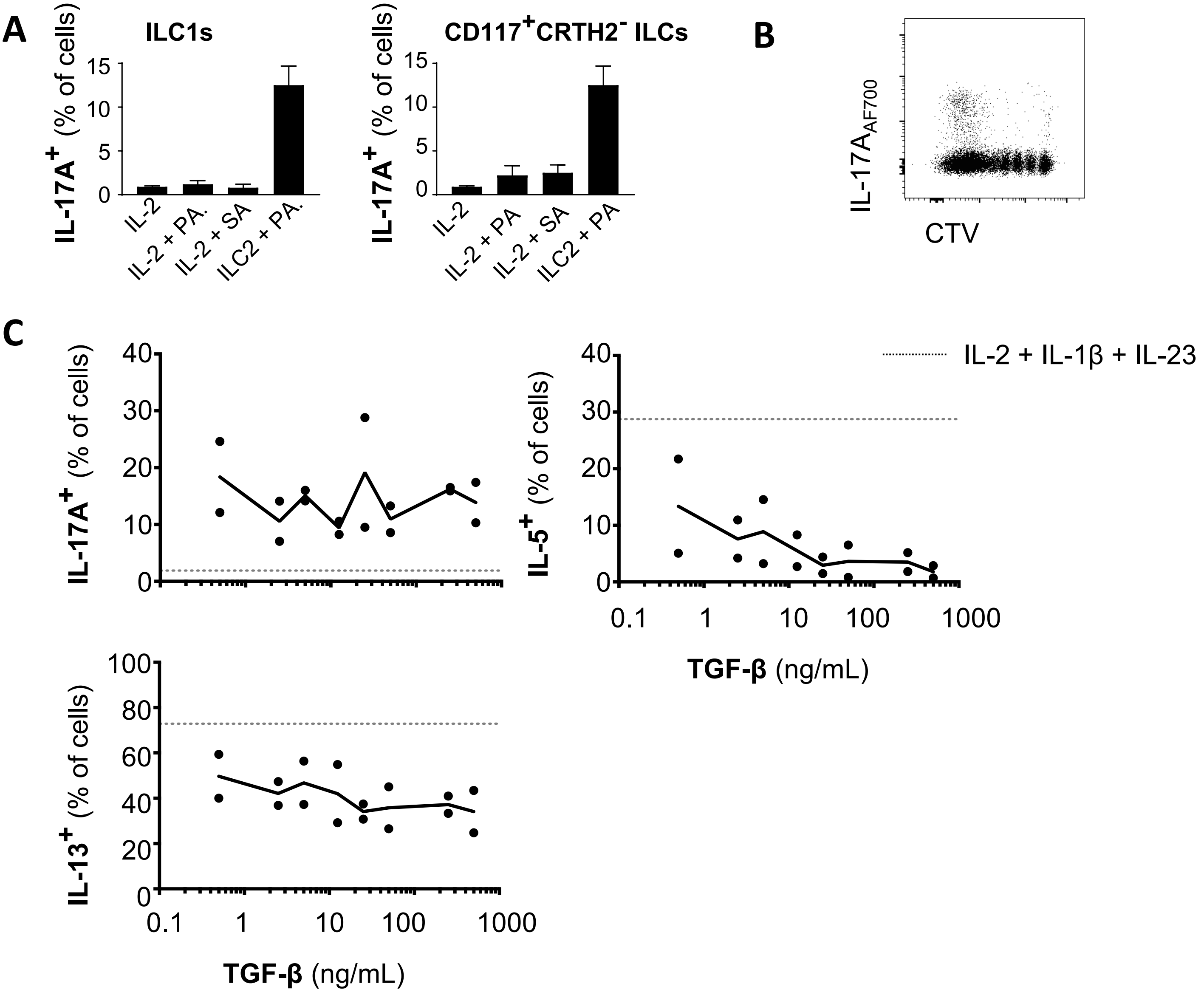

Supplementary Figure 2. Nasal epithelial derived cytokines drive ILC2 plasticity. (A) Quantification of IL-17A-producing blood-derived ILC subsets exposed to SA or PA for 7 days in the presence of NP epithelium and IL-2 after PMA/ionomycin stimulation (n = 5). Error bars represent S.E.M.. (B) Representative flow cytometric analysis of intracellular IL-17A production and proliferation of blood ILC2s upon culture for 5 days with IL-2 (20 U/mL), IL-1β, IL-23, and TGF-β (all 20 ng/mL, n = 3). (C) Quantification of IL-17, IL-5 and IL-13$^+$ ILC2s after culture of blood ILC2s for 7 days with IL-2, IL-1β, IL-23, and increasing doses of TGF-β (n = 2). (D) Representative flow cytometric analysis of IL-5-, IL-13-, and IL-17A-producing blood ILC2s co-cultured with OP-DL1 and stimulated for 9 days with IL-2/IL-7; IL-2/IL-7/IL-33/TSLP; IL-2/IL-7/IL-1β/IL-23/TGF-β and re-stimulated with PMA/ionomycin (n = 6). (E) Quantification of IL5- and IL-13-producing blood-ILC2 co-cultured with OP9 or OP9-DL1 and exposed to cytokines (n = 6). (F) Quantification of IL-17A$^+$ ILC2s after culture of blood ILC2s for 7 days with IL-2, IL-1β, IL-23, TGF-β with and without IL-6 (n = 6). (G) Flow cytometric analysis of IL-5 and IL-17A-producing ILC2s exposed to IL-2, as a control condition for the experiments shown in Figure 2A and 2B. *p < 0.05, **p < 0.01, ***p<0.001 as determined by one way ANOVA.

Supplementary Figure 2. (related to Figure 2)

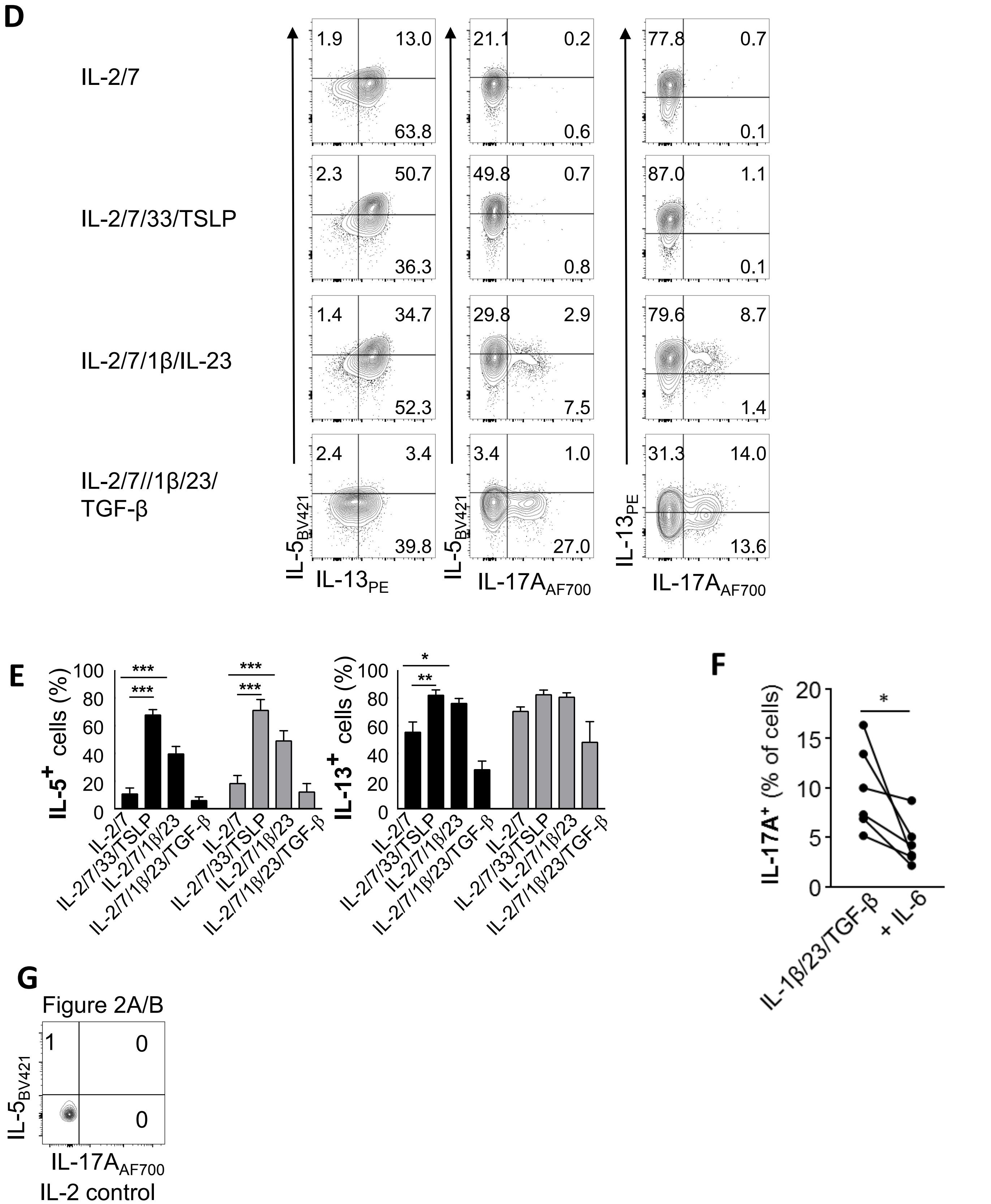

Supplementary Figure 3. (related to Figure 3)

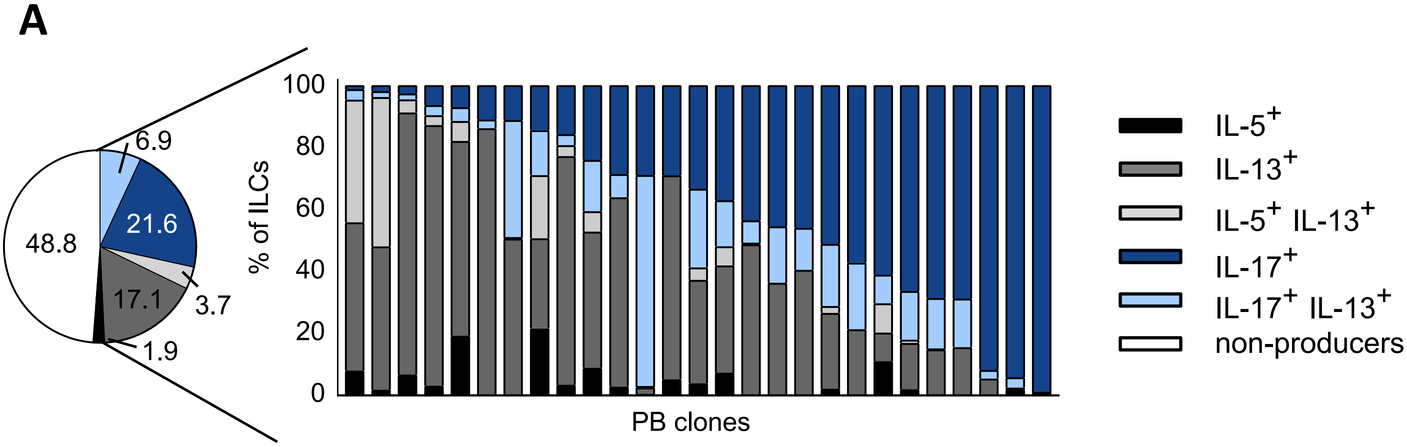

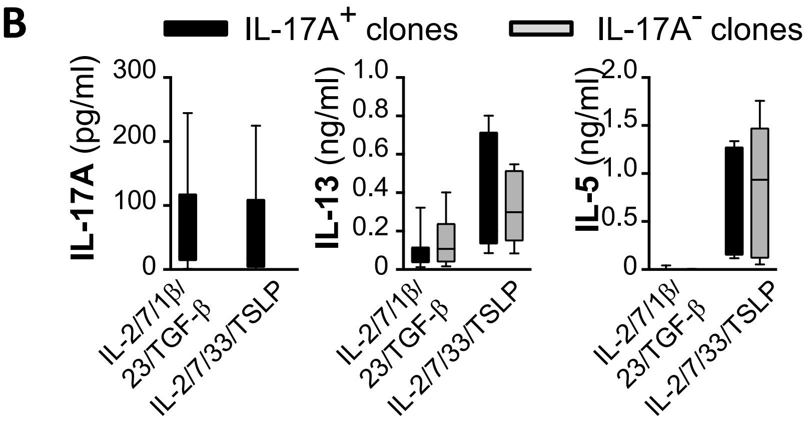

Supplementary Figure 3. ILC2s give rise to IL-17 producing cells at a clonal level.
(A) Single ILC2s from blood were sorted by FACS into 96-well round bottom plates pre-seeded with OP9-DL1 and stimulated with IL-2, IL-7, IL-1β, IL-23, and TGF-β. After 14-21 days, cultures were analysed for intracellular cytokine production (IL-5, IL-13, and IL-17A) after PMA/ionomycin stimulation. (B) Quantification of concentration of IL-5, IL-13, and IL-17A secreted by IL-17A-producing or non-producing blood ILC2 clones from (**A**) (n = 8). The error bars represent S.E.M.. Data are summarized from three independent experiments (A).

Supplementary Figure 4. (related to Figure 5)

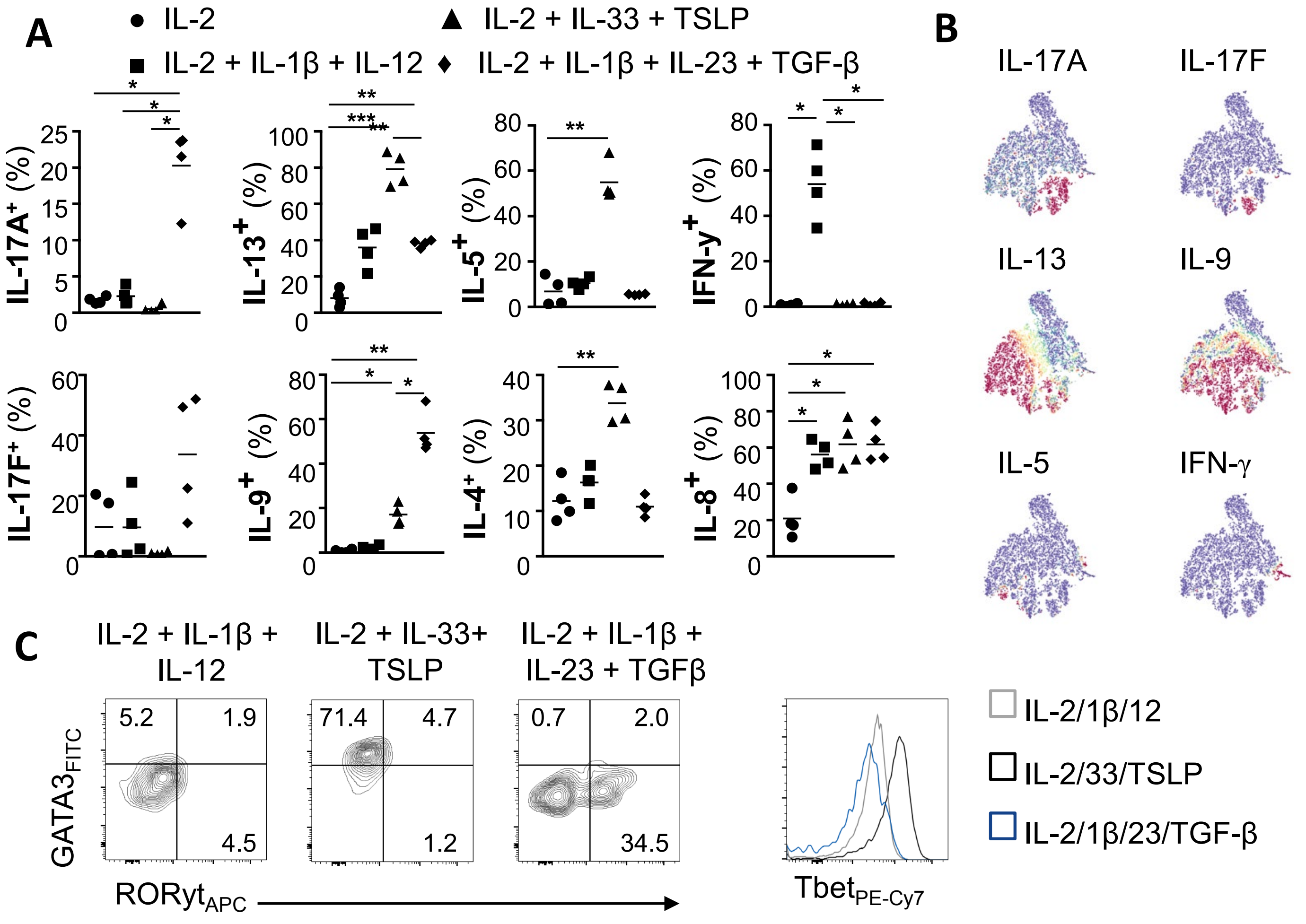

Supplementary Figure 4. The tissue cytokine balance modulates ILC2 plasticity.
(A) Intracellular cytokine production by blood ILC2s exposed to different cytokines for 5-7 days. Each symbol represents an individual donor and the horizontal lines represent the mean (n = 4). (B) t-SNE analysis on three different blood samples showing cytokine expression by blood ILC2s exposed to IL-2, IL-1β, IL-23, and TGF-β. (C) Representative flow cytometric plots of transcription factors expression in blood ILC2s (n = 6). (D) Heatmap of Pearson's coefficients of correlation for the 25% of probes with the greatest difference in expression among all samples. Yellow indicates the highest correlation. (E) Vulcano plot comparing ILC2s stimulated with IL-2, IL-1β, IL-23, and TGF-β with freshly isolated ILC2s. (F) Isotype or unstimulated control condition corresponding to Figure 4B and 4F. *p < 0.05, **p < 0.01, ***p<0.001 as determined by one way ANOVA.

Supplementary Figure 4. (related to Figure 5)

D

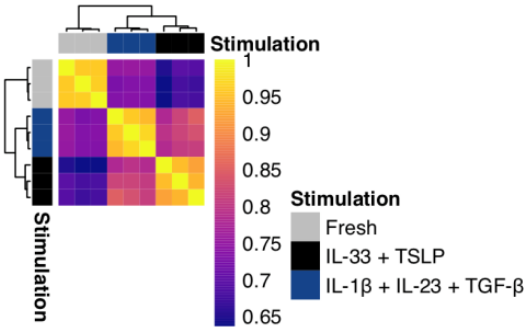

E IL-2/1β/23/TGF-β versus fresh

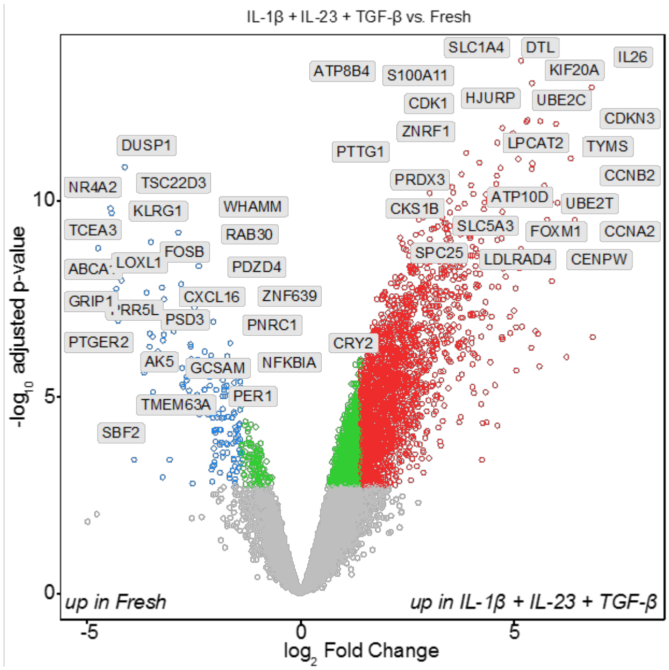

F

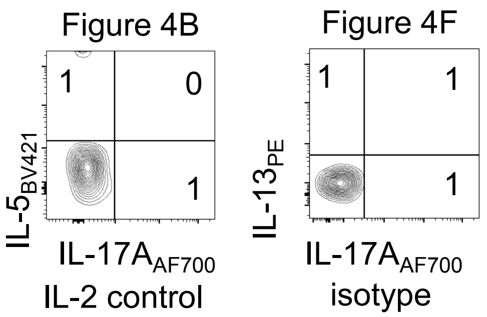

Supplementary Figure 5. (related to Figure 5)

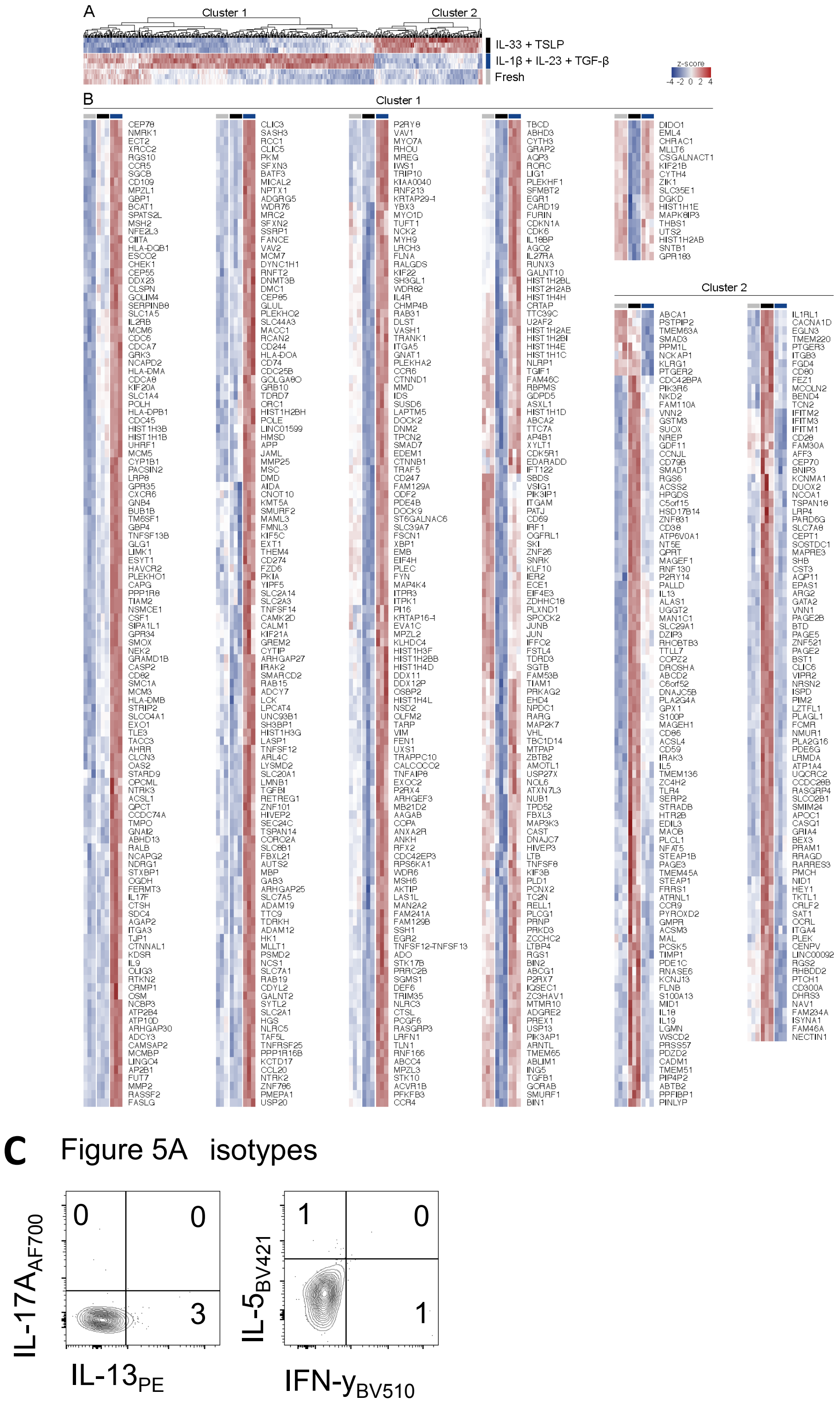

Supplementary Figure 5. Transcriptome analysis of ILC2 plasticity

(A) Unbiased clustering showing clusters of the number of genes with significant different expression between blood ILC2s stimulated with IL-2, IL-1β, IL-23, and TGF-β as compared to ILC2s cultured with IL-2, IL-33, and TSLP. (B) Heatmap of genes in the clusters identified in (**A**). Z-scores (row-normalized) are shown. (C) Isotypes corresponding to Figure 5A.

Figure S6. (related to Figure 6)

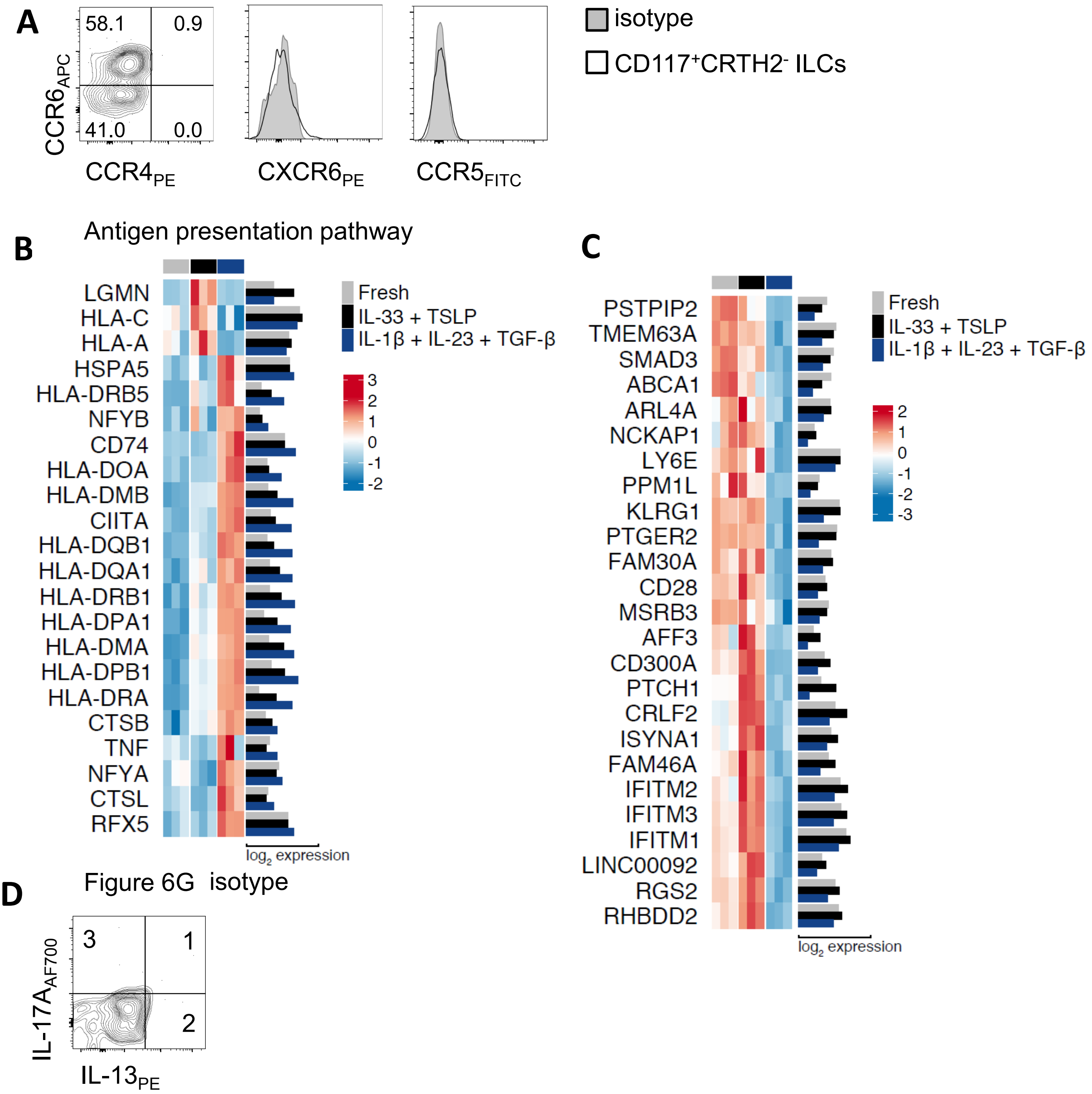

Supplementary Figure 6. ILC2 stimulations result in unique expression patterns
(A) Representative flow cytometric analysis of CCR4, CCR5, CCR6, and CXCR6 expression on peripheral blood CD117$^+$ CRTH2$^-$ ILCs. Data are representative of 3 different donors. (B) Heatmap of genes significantly different expressed in ILC2s upon stimulation with IL-2, IL-1β, IL-23, and TGF-β as compared to ILC2s cultured with IL-2, IL-33, and TSLP that are related to the antigen presentation KEGG pathway. (C) Heatmap of genes that are significantly lower expressed in blood ILC2s cultured with IL-2, IL-1β, IL-23, and TGF-β as compared to freshly isolated ILC2s and ILC2s cultured with IL-2, IL-33, and TSLP. Z-scores (row-normalized) are shown. (D) Isotypes corresponding to Figure 6G.

Figure S7. (related to Figure 7)

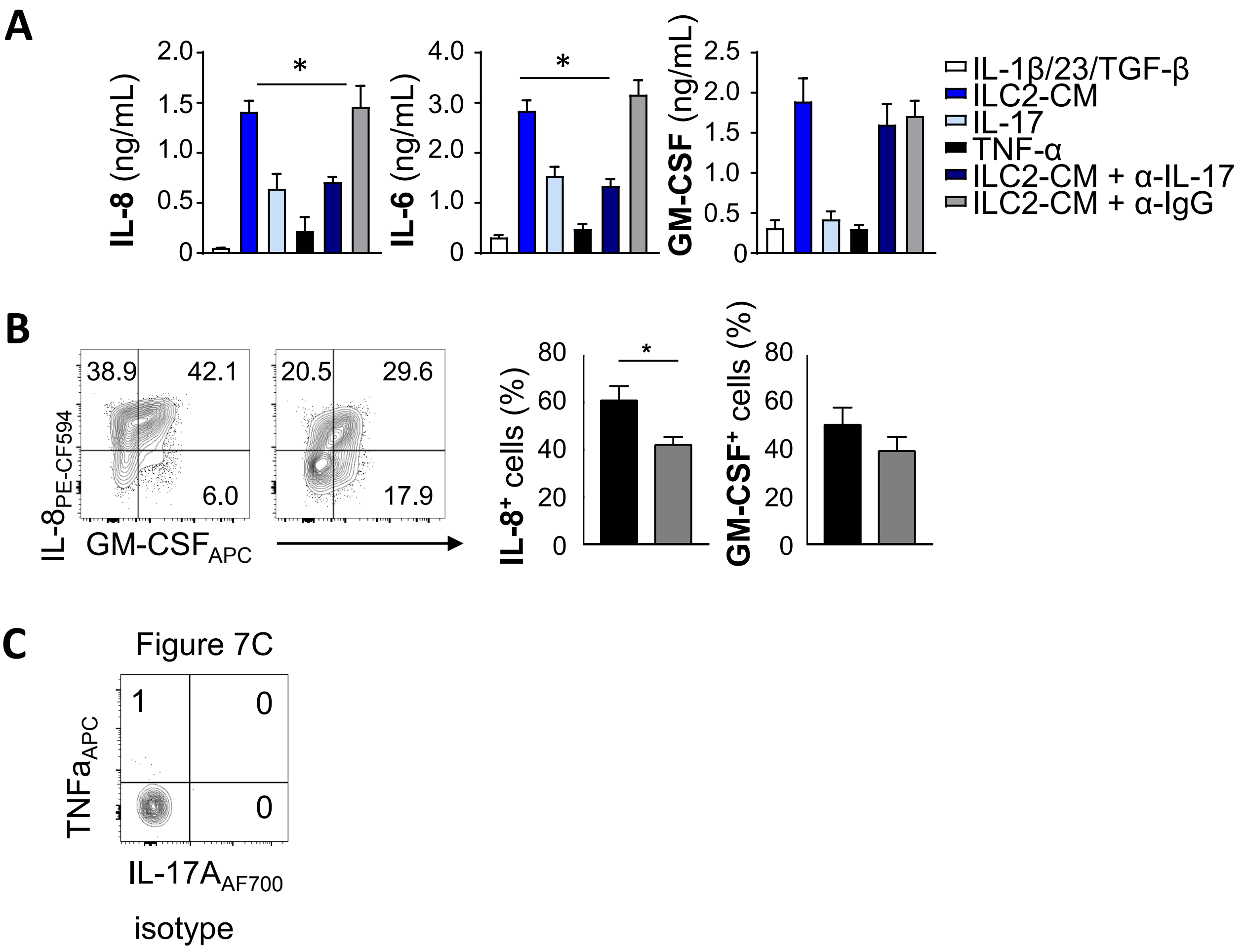

Supplementary Figure 7. Transdifferentiated ILC2s enhance neutrophil recruitment via epithelium (A) Quantification of IL-8, IL-6, and GM-CSF produced by NCI-H292 cells exposed for 48 hours to IL-1β, IL-23, TGF- β; IL-17; TNF-α; or in the presence or absence of IL-17-blocking antibody or isotype control to conditioned medium of ILC2s, that were stimulated with IL-2, IL-1β, IL-23, and TGF-β for 5-7 days (n = 3). (B) Flow cytometry analysis and quantification of intracellular expression of IL-8 and GM-CSF after PMA/ionomycin in blood ILC2s exposed to IL-2, IL-1β, IL-23, and TGF-β for 5-7 days. (C) Isotypes corresponding to Figure 7C. *$p < 0.05$ as determined by one way ANOVA. Data are presented as mean ± S.E.M.

Figure S8. (related to Figure 8)

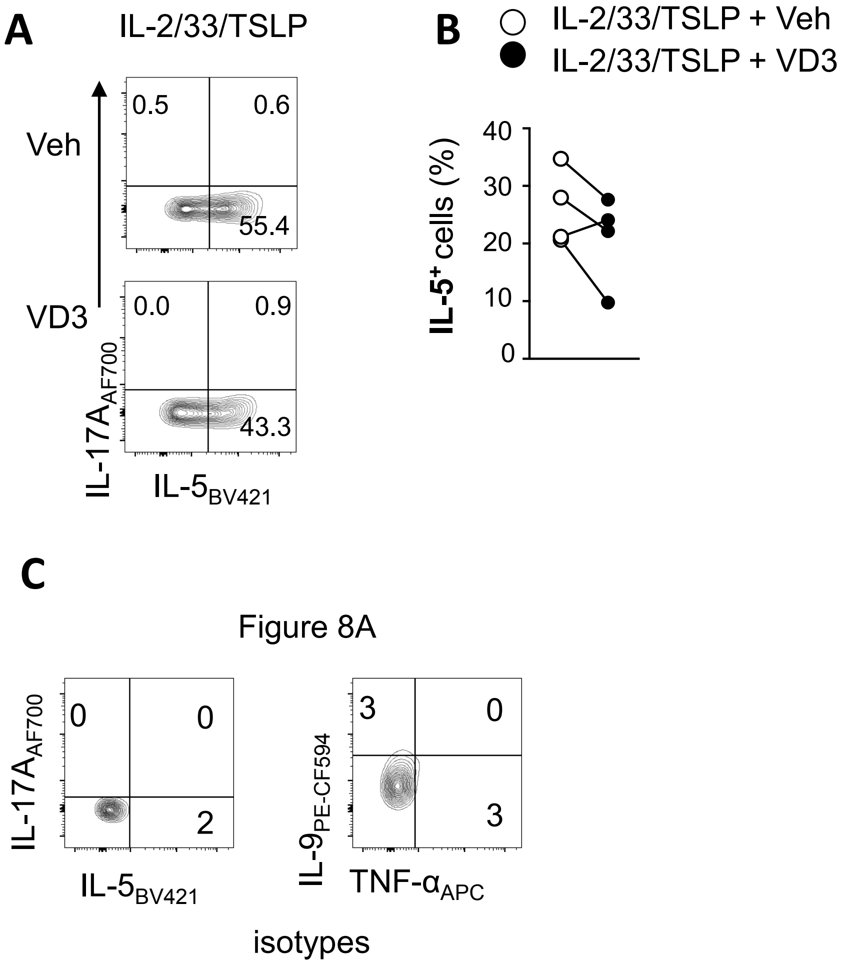

Supplementary Figure 8. Vitamin D3 prevents ILC2 conversion by dampening IL-23 signalling pathway (A) Representative flow cytometric analysis of intracellular IL-5 and IL-17A production by blood ILC2s after culture for 7 days with IL-2, IL-33, and TSLP in the presence of VD3 or vehicle control. (B) Quantification of IL-5-producing cells after culture as in (A) (n = 3). (C) Isotypes corresponding to Figure 8A. Data are presented as individual values of three independent experiments.

Supplementary Table 1

GAPDH	forward	GTC TCC TCT GAC TTC AAC AGC G
	reverse	ACC ACC CTG TTG CTG TAG CCA A
B-actin	forward	CAC CAT TGG CAA TGA GCG GTT C
	reverse	AGG TCT TTG CGG ATG TCC ACG T
GATA3	forward	ACC ACA ACC ACA CTC TGG AGG A
	reverse	TCG GTT TCT GGT CTG GAT GCC T
RORC	forward	AAT CTG GAG CTG GCC TTT CA
	reverse	CTG GAA GAT CTG CAG CCT TT
TBX21	forward	ATT GCC GTG ACT GCC TAC CAG A
	reverse	GGA ATT GAC AGT TGG GTC CAG G
IL4A	forward	GAC CTG GAG CAA CCC GTA TC
	reverse	GAT GCG GAG GGA GGG TTC TA
IL17A	forward	GAAGGCAGGAATCACAATC
	reverse	GCCTCCCAGATCACAGA
IL1R1	forward	GTGCTTTGGTACAGGGATTCCTG
	reverse	CACAGTCAGAGGTAGACCCTTC
TGFBR1	forward	CTGCCTGGCTAGAGAAGCACAA
	reverse	TCATACAGGTCTGAGCGTGTGG
CCR4	forward	GGG GTC ATC ACC AGT TTG
	reverse	TCT TCA CCG CCT TGT TCT
CCR6	forward	TTCAGCGATGTTTTCGACTCC
	reverse	GCAATCGGTACAAATAGCCTGG
CXCR6	forward	GACTATGGGTTCAGCAGTTTCA
	reverse	GGCTCTGCAACTTATGGTAGAAG
CCR5	forward	TTCTGGGCTCCCTACAACATT
	reverse	TTGGTCCAACCTGTTAGAGCTA

Supplementary Table 1. List of primers used in this study.
