# Supplementary material for: IL-1β, IL-23, and TGF-β drive plasticity of human ILC2s towards IL-17-producing ILCs in nasal inflammation
Source: Nat Commun. 2019 May 14;10:2162. doi: 10.1038/s41467-019-09883-7 (PMC6517442; doi:10.1038/s41467-019-09883-7)
Supplement: Supplementary file 2 — Description of Additional Supplementary Files [file 41467_2019_9883_MOESM2_ESM.pdf]

Description of Additional Supplementary Files

File Name: Supplementary Data 1
Description: Raw data of microarray analysis
