# Supplementary material for: IL-1β, IL-23, and TGF-β drive plasticity of human ILC2s towards IL-17-producing ILCs in nasal inflammation
Source: Nat Commun. 2019 May 14;10:2162. doi: 10.1038/s41467-019-09883-7 (PMC6517442; doi:10.1038/s41467-019-09883-7)
Supplement: Supplementary file 4 — Reporting Summary [file 41467_2019_9883_MOESM4_ESM.pdf]

nature research

Corresponding author(s): Hergen Spits

Last updated by author(s): Mar 10, 2019

Reporting Summary

Nature Research wishes to improve the reproducibility of the work that we publish. This form provides structure for consistency and transparency in reporting. For further information on Nature Research policies, see Authors & Referees and the Editorial Policy Checklist.

Statistics

For all statistical analyses, confirm that the following items are present in the figure legend, table legend, main text, or Methods section.

n/a	Confirmed	
☐	☒	The exact sample size (*n*) for each experimental group/condition, given as a discrete number and unit of measurement
☐	☒	A statement on whether measurements were taken from distinct samples or whether the same sample was measured repeatedly
☐	☒	The statistical test(s) used AND whether they are one- or two-sided<br>*Only common tests should be described solely by name; describe more complex techniques in the Methods section.*
☐	☒	A description of all covariates tested
☐	☒	A description of any assumptions or corrections, such as tests of normality and adjustment for multiple comparisons
☒	☐	A full description of the statistical parameters including central tendency (e.g. means) or other basic estimates (e.g. regression coefficient) AND variation (e.g. standard deviation) or associated estimates of uncertainty (e.g. confidence intervals)
☐	☒	For null hypothesis testing, the test statistic (e.g. *F*, *t*, *r*) with confidence intervals, effect sizes, degrees of freedom and *P* value noted<br>*Give P values as exact values whenever suitable.*
☒	☐	For Bayesian analysis, information on the choice of priors and Markov chain Monte Carlo settings
☒	☐	For hierarchical and complex designs, identification of the appropriate level for tests and full reporting of outcomes
☐	☒	Estimates of effect sizes (e.g. Cohen's *d*, Pearson's *r*), indicating how they were calculated

Our web collection on statistics for biologists contains articles on many of the points above.

Software and code

Policy information about availability of computer code

Data collection	Flow cytometry data were acquired with FACSDiva 8.0.1 software. Gene expression was obtained from Clariom D microarray.
Data analysis	FlowJo V10, GraphPad Prism v.7.05 and MS Excel 2010 were used to analyze the data.<br>For microarray: all data analysis was performed in RStudio; data were visualized using ggplot2 and pheatmap R-packages.

For manuscripts utilizing custom algorithms or software that are central to the research but not yet described in published literature, software must be made available to editors/reviewers. We strongly encourage code deposition in a community repository (e.g. GitHub). See the Nature Research guidelines for submitting code & software for further information.

Data

Policy information about availability of data

All manuscripts must include a data availability statement. This statement should provide the following information, where applicable:

- Accession codes, unique identifiers, or web links for publicly available datasets
- A list of figures that have associated raw data
- A description of any restrictions on data availability

The datasets generated during and/or analysed during the current study are available from the corresponding author on reasonable request. Raw data files for figure 1A, C-E, 2B-H, 3A, C-E, 4A, C, E, G, H, J, K, 5B, C, 6E, 7A, B, D-F, 8B, C, E, F are available in the SOURCE DATA FILE.

Field-specific reporting

Please select the one below that is the best fit for your research. If you are not sure, read the appropriate sections before making your selection.

☒ Life sciences ☐ Behavioural & social sciences ☐ Ecological, evolutionary & environmental sciences

For a reference copy of the document with all sections, see nature.com/documents/nr-reporting-summary-flat.pdf

Life sciences study design

All studies must disclose on these points even when the disclosure is negative.

Sample size	The sample size is limited by the availability of the patient material that we receive from the operating room from the ENT clinic. The sample size we considered sufficient to support our statements in the text was determined by the statistical tests (we describe which tests were used in the main text of the manuscript).
Data exclusions	Does not apply
Replication	Each experiment was perfored at least three times. and with three different sample donors, in most cases more than three. Only the data that could be reproduced are demostrated in this study.
Randomization	n/a
Blinding	n/a

Reporting for specific materials, systems and methods

We require information from authors about some types of materials, experimental systems and methods used in many studies. Here, indicate whether each material, system or method listed is relevant to your study. If you are not sure if a list item applies to your research, read the appropriate section before selecting a response.

Materials & experimental systems

n/a	Involved in the study	
☐	☒	Antibodies
☐	☒	Eukaryotic cell lines
☒	☐	Palaeontology
☒	☐	Animals and other organisms
☐	☒	Human research participants
☒	☐	Clinical data

Methods

n/a	Involved in the study	
☒	☐	ChIP-seq
☐	☒	Flow cytometry
☒	☐	MRI-based neuroimaging

Antibodies

Antibodies used	Biolegend: anti-human CD1a FITC (HI149), Cat#300104; anti-human CD3 FITC (OKT3), Cat#317306; anti-human CD14 FITC (HCD14), Cat#325604; anti-human CD16 FITC (3G8), Cat#555406; anti-human CD19 FITC (HIB19),Cat#302206; anti-human CD34 FITC (581), Cat#2317520; anti-human CD94 FITC (DX22), Cat#305504; anti-human CD123 FITC (6H6), Cat#306014; anti-human FcER1α FITC (AER37), Cat#2273040; anti-human TCRαβ FITC (IP26), Cat#306706; anti-human TCRγδ FITC (B1), Cat#331208; anti-human BDCA2 FITC (201A), Cat#354208; anti-human IL-17F FITC (Poly5166),Cat#3183029; anti-human CD161 PE (HP-3G10), Cat#339904; anti-human NKp44 PE (P448),Cat#325108; anti-human IL-9 PE (MH9A4),Cat#507605; anti-human HLA-DR PE (L243),Cat#307605; anti-human IL-5 PE (JES1-39D107), Cat#500904; anti-human CD45 AF700 (HI30),Cat#304024; anti-human CD3 AF700 (UCHT1),Cat#300424; anti-human IL-17A AF700 (BL168),Cat#512318; anti-human CD161 BV421 (HP-3G10),Cat#339914; anti-human IL-5 BV421 (JES1-39D10),Cat#504311; anti-human STAT3 Phospho BV421(13A3-1), Cat#651009; anti-human IFNγ BV510 (4S.B3),Cat#502544; anti-human CRTH2 PE-CF594 (BM16),Cat#350126; anti-human CD45 PE-CF594 (HI30), Cat#304052; anti-human IL-13 APC (JES10-5A2),Cat#501903; anti-human Siglec-8 APC (7C9),Cat#347105; anti-human CD16 PE-Cy7 (38G), Cat#302015;<br>eBioscience: anti-human T-bet PE-Cy7 (4B10), Cat#12582582; anti-human GATA3 PE (TWAJ), Cat#12996642; anti-human KLRG1 APC (13F12F2),Cat#17948842<br>Beckman Dickinson: anti-human CD127 PE-Cy7 (R34.34),Cat#A64618; anti-human CD117 PE-Cy5 (104D2D1),Cat#B96754; anti-human CRTH2 AF647 (BM16),Cat#558042; anti-human CD45 APC-Cy7 (2D1),Cat#368518; anti-human CD94 PerCP-Cy5.5 (HP-3D9),Cat#305514; anti-human RoRγT AF647 (Q21-559),Cat#563620; anti-human Smad2/Smad3 AF647 (O72-670), Cat#562696
Validation	all antibodies were purchased directly from the manufacturers, no further validation was performed.

Eukaryotic cell lines

Policy information about cell lines

Cell line source(s)	NCI-H292 [H292] (ATCC® CRL-1848™)
Authentication	The cells were purchased from ATCC, no authentication was performed.
Mycoplasma contamination	This cell line tested negative for mycoplasma contamination.
Commonly misidentified lines (See ICLAC register)	n/a

Human research participants

Policy information about studies involving human research participants

Population characteristics	For the purpose of this study, no specific recruitment of patients was performed. Tissue samples used in this study were waste material from patients who underwent a surgery at the ENT clinics at the Academic Medical Center in Amsterdam, The Netherlands or in Leuven, Belgium.<br>Uninflamed nasal inferior turbinates were obtained from patients who underwent corrective surgery for hypertrophy with or without septoplasty. Nasal polyps were obtained from CRSwNP patients with or without cystic fibrosis undergoing endoscopic sinus surgery.<br>We used tissue material of 31 patients, aged 18-62/ male/female ratio of 52/48, 13 of them were diagnosed with CRSwNP, 7 with CFwNP, and 11 with deviated septum.
Recruitment	For the purpose of this study, no specific recruitment of patients was performed. Tissue samples were waste material from patients who underwent a surgery at the ENT clinics at the Academic Medical Center in Amsterdam, The Netherlands or in Leuven, Belgium.
Ethics oversight	Medical Ethical Committee of the Academic Medical Center in Amsterdam, the Netherlands.

Note that full information on the approval of the study protocol must also be provided in the manuscript.

Flow Cytometry

Plots

Confirm that:

☒ The axis labels state the marker and fluorochrome used (e.g. CD4-FITC).

☒ The axis scales are clearly visible. Include numbers along axes only for bottom left plot of group (a 'group' is an analysis of identical markers).

☒ All plots are contour plots with outliers or pseudocolor plots.

☒ A numerical value for number of cells or percentage (with statistics) is provided.

Methodology

Sample preparation	Peripheral blood ILC isolation: prior to sorting, cells were first enriched by labeling with PE-conjugated anti-CD161, followed by anti-PE microbeads (Miltenyi) according to the manufacturer's instructions. Sorting from nasal tissues was performed without pre-enrichment. Cells were sorted as Lin- (CD1a, CD3, CD14, CD16, CD19, CD34, CD94, CD123, BDCA2, FcεRI, TCRαβ, TCRγδ), CD45+, CD127+, and CD161+. ILC2s were sorted as CRTH2+ cells, NKp44+ ILC3s as CRTH2-CD117+NKp44+ cells, and NKp44- ILC3s as CRTH2-NKp44-CD117+ cells. Cells were sorted on a FACSAria (BD) to a purity >99% either in bulk or as single cells.<br>For FACS analysis, cells were stained with antibodies for 20 minutes at 4°C in PBS. Epithelial cells were obtained by incubating single cell suspension with anti-EpCAM MicroBeads (Miltenyi Biotec) and a positive selection on a magnetic column. For experiments involving intracellular cytokine staining, cells were stimulated with PMA (10 ng/ml; Sigma) plus Ionomycin (500 nM; Merck) in the presence of Golgi Plug (BD) for 3 h at 37°C. Afterwards cells were fixed, permeabilized, and stained using the Foxp3/Transcription Factor Staining Buffer Kit (ThermoFisher Scientific). For detection of pSTAT3 and pSMAD2/3 samples were stimulated for 10 min. Cells were fixed (BD Cytofix fixation buffer, BD Biosciences) for 15 min at 4°C and permeabilized (BD Phosflow Perm Buffer III) for 30 min on ice. Afterwards, cells were stained with antibodies for 30 min at room temperature.
Instrument	Samples were acquired on LSRFortessa or FACSCanto II (BD Biosciences).
Software	Data were acquired using FACSDiva 8.0.1 software and analyzed with FlowJo V10.
Cell population abundance	Cells were sorted on a FACSAria (BD) to a purity >99% either in bulk or as single cells.<br>10,000 events were originally collected from which positively-gated cells showed 95-99% purity

Gating strategy	All samples were FSC-A and SSC-A gated, followed by FSC-A/FSC-H gating to select singlet cells. Subsequent relevant gating strategy is descibed in detail in the Fig 1B.

☒ Tick this box to confirm that a figure exemplifying the gating strategy is provided in the Supplementary Information.
